# Critical delay factors for construction projects in Central Aceh District, Indonesia

**DOI:** 10.12688/f1000research.110024.1

**Published:** 2022-04-29

**Authors:** Anita Rauzana, Aghnia Zahrah, Wira Dharma

**Affiliations:** 1 Department of Civil Engineering, Universitas Syiah Kuala, Banda Aceh, 23111, Indonesia; 2 Department of Architecture and Planning, Universitas Syiah Kuala, Banda Aceh, 23111, Indonesia; 3 Faculty of Mathematics and Natural Sciences, Universitas Syiah Kuala, Banda Aceh, 23111, Indonesia

**Keywords:** delay factors, project management, risk management, construction industry, schedule delays

## Abstract

**Background:** Construction development in Indonesia is growing rapidly, especially in Central Aceh District. Construction projects have distinctive characteristics and are very complex, so that risk events can have a serious impact on the viability of the project. A lack of attention to the risks faced will affect project implementation by creating delays, resulting in losses. The purpose of this study was to (1) identify the risk factors that cause delays in construction projects and (2) determine those particular risk factors that have a greater influence on construction projects. The location of this research was Central Aceh District, Indonesia.

**Methods:** The data in this study were primary data in the form of a questionnaire and secondary data obtained from the literature related to this particular type of research. Questionnaires were distributed to respondents, namely contractor companies located in the Central Aceh District. The questionnaires were distributed to determine respondents' opinions about the level of influence of risk factors causing project delays. We used a validity test, reliability test, and descriptive analysis for data processing.

**Results: **Based on the results of the study from 47 respondents, the “very high influence” category (Mode=5) for the tool malfunction factor was chosen by 21 respondents (44.68%), cost estimation inaccuracy by 20 respondents (42.55%), increased work costs by 22 respondents (46.81%), implementation of new technologies by 25 respondents (53.19%), details, accuracy and conformity to specifications that are not appropriate by 20 respondents (42.55%), worker quarrels by 20 respondents (42.55%), poor project planning and management by 22 respondents (46.81%), poor condition at locations and accessibility difficulty by 20 respondents (42.55%).

**Conclusions: **Of the 80 risk factors that caused project delays, eight risk factors were found to have a very high influence on the implementation of construction projects in Central Aceh District.

## Introduction

The Central Aceh Regency is one of the regions in Aceh Province, Indonesia, experiencing rapid growth. This can be observed in the number of construction projects currently underway. ^1^ The implementation of construction projects in the Central Aceh Regency often experiences failures and delays, ^1^ which can cause project losses. Construction projects are dynamic and consist of limited resources. A complex project can cause high-risk and uncertain events that can cause delays and cost overruns on projects, ^2^ thus allowing for uncertainty in the implementation process, which leads to various types of risks that ultimately cause losses to the parties involved in the construction project and affect the achievement of the desired goal. Risk is a condition in which there is a possibility of gain/loss, ^3^ with losses, such as cost losses, injuries, and delays caused by uncertainty during project implementation. One of the most influential risk factors is changing the order. ^4^ Delays in the implementation of projects are among the risks that often occur in the implementation of construction projects, especially in developing countries. ^5^^–^^8^ Project delays and cost overruns can harm projects. ^9^

An increase in fuel prices can cause cost increases, losses, and delays in construction projects. ^6^^,^^10^ There are five causes for a project loss: (1) improper planning and scheduling, (2) many changes to orders by clients, (3) incompetent site management and supervision, (4) inexperienced subcontractors, and (5) poor contractor finances. ^11^ Experienced contractors can accelerate a project schedule. ^12^ The most influential risk factor for projects in Jordan (the Middle East) is poor soil/site conditions in construction projects. ^13^

Previous research has shown that delays in project implementation can lead to cost overruns. ^14^ Delays affect planning and control, ^15^ especially during project implementation. ^16^ Project delays can lead to losses, legal problems, and contract termination. ^17^^,^^18^ The contractor suffers losses owing to cost overruns. For example, in Nigeria, cost overruns and delays are frequent factors affecting projects. ^19^ It is important to apply risk management to avoid project failure because construction projects are complex and involve many risks. ^20^ Project risk is defined as an unforeseen event or situation that can harm a project. ^21^ Risk management is an important process for achieving project objectives. ^22^^,^^23^ Identifying, assessing, and managing construction project risks is indispensable for risk management. A successful project is time- and cost-effective, and has good construction quality. ^24^ Managing risk is an important mechanism in the construction sector, which is performed to obtain project objectives in the form of cost, time, safety, and good quality ^25^; the most influential risk factor is material. ^26^

Development projects globally often involve considerable risk. Inflation causes delays and losses. ^27^ Risks can affect the time, cost, quality, and performance of a construction project. ^28^ Time risk affects project costs. Project risk management aims to increase profits and reduce losses. ^29^

For construction projects, overtime or delays are common during project implementation. Time delays can be described as events or interruptions that result in a project not being completed within the time specified in the contract. Defining delays as actions or activities that increase the time required by the contractor to conduct the project is referred to as time contingency. ^30^

Only 30% of Saudi Arabian construction projects implemented require an average additional time of approximately 10–30%, where there are nine main groups of risk factors causing delays: costs, resources, contracts, schedule, government relations, personnel, planning, equipment, and environmental factors. Funding delay is the most important delay factor. ^31^ In project implementation, the contractor company does not know the risk of project delays. Therefore, to avoid losses and delays in construction projects, research is needed to identify and analyze the factors causing delays in construction projects, particularly in Indonesia and the Central Aceh District, given the complex conditions of the district, including socio-cultural diversity, high inflation rates, low public education, frequent disasters, community economy weakness, geographic location, social and political conflicts, and economic crises. ^32^

Risk identification is conducted by collecting all information related to activities and analyzing it to find every possible risk that could result in a loss. Risk identification can be performed using several techniques. ^33^ Identifying risks in a project consists in compiling (1) a list of risks that can cause losses, (2) a list of potential losses, and in this checklist compiling (3) a list of losses and (4) a ranking of losses occurring, and then (5) classifying losses. Project delays also occur owing to work accidents. ^34^ The type of soil and rock at the project site is one of the main risk factors for project delay. ^35^

## Methods

### Questionnaire design

The primary data in this study was questionnaire data; the questionnaire was distributed to 47 respondents and contained 80 questions about project delays. Secondary data were obtained from studies in the literature such as journals, books, and other literature related to this research, as well as data about contractor companies obtained from the National Construction Services Association. The distribution of questionnaires aimed to determine the level of influence of risk factors causing project delays; a closed questionnaire was used, where answer choices had been determined in advance, and respondents were given the opportunity to choose the most appropriate answer. ^36^ For data processing, we used a validity test, a reliability test, and descriptive analysis.

The questionnaire was composed of two parts: questionnaire A and questionnaire B. Questionnaire A concerned the characteristics of respondents, and questionnaire B concerned the level of influence of factors causing project delays. Assessment of the level of influence of 80 project delay risk factors was carried out using a Likert scale, which consists of five points as defined in previous studies ( *e.g.*, References 31, 37, 38). The Likert scale has previously been used to measure the perceptions of respondents about social events ^39^^–^^41^ and can be seen in Table 1.

**Table 1.** Likert scale.

No.	Category	Score
1	Very high influence	5
2	High influence	4
3	Medium influence	3
4	Low influence	2
5	Very low influence	1

### Data collection

The data collected for this research were questionnaire data, from questionnaire tools distributed to respondents, namely contractor companies located in Central Aceh District. The collection of data was carried out over two months by the researchers. This study used probability sampling, namely simple random sampling in distributing questionnaires. Simple random sampling technique is a technique consisting in taking samples randomly from members of the population. ^42^ The targeted respondents were contractors from the Central Aceh Regency, which has a population of 53 contractor companies. The experimental procedures were approved by the Institutional Review Board at Syiah Kuala University (IRB protocol number 99). All of these experimental methods were carried out in accordance with the regulations of the Institutional Review Board of Syiah Kuala University in Indonesia, and all participants gave their informed consent. The total sample size was 47 companies, calculated from the total population with an inaccuracy allowance of 5%, then by using the Slovin formula. ^43^ Data collection was performed by distributing questionnaires to respondents directly.

$$n=\frac{N}{1+\left(N\;x\;e^{2}\right)}$$ (1)

Table 2 shows that based on the identification of risk factors for delay, there were 80 causes of construction project delays, which were categorised into seven main factors.

**Table 2.** Factors causing project delays.

No	Description of causes	Category
1	Increase in material prices	Material
2	Delay in material delivery	Material
3	Material theft	Material
4	Substandard material quality	Material
5	The volume and type of material is not appropriate	Material
6	Damage during shipping and storage material	Material
7	Limited material storage space	Material
8	Supplier cannot fulfil material order	Material
9	Poor material planning & management	Material
10	Waste material handling	Material
11	Small equipment capacity (small production)	Equipment
12	Equipment misplacement	Equipment
13	Delay in equipment mobilization	Equipment
14	Incomplete equipment	Equipment
15	Tool malfunction	Equipment
16	Negligence in checking the condition of the equipment	Equipment
17	Productivity and efficiency decreased	Equipment
18	Additional equipment rental costs	Equipment
19	Fuel scarcity	Equipment
20	Difficult access for heavy equipment to be used during the execution of the project site	Equipment
21	Poor equipment planning & management	Equipment
22	High equipment maintenance cost	Equipment
23	Do not understand the procedure for using equipment	Equipment
24	Equipment not in accordance with condition	Equipment
25	Ownership of rental equipment	Equipment
26	Ownership of the lease-purchase equipment	Equipment
27	Ownership of proprietary equipment	Equipment
28	Owner does not pay on time	Financial
29	Cost estimation inaccuracy	Financial
30	Did not predict unexpected costs	Financial
31	Delay penalty	Financial
32	Increased costs due to environmental safeguards	Financial
33	Increased work costs	Financial
34	Inefficient budgeting	Financial
35	Availability of cash	Financial
36	Availability of project financing sources (debtors) banks/third parties	Financial
37	Profit target	Financial
38	Unofficial charges	Financial
39	Financial constraints on the contractor	Financial
40	Investors bankruptcy	Financial
41	Incompatibility of the use of costs with the progress of work	Financial
42	Inaccurate construction method causes errors during project	Construction method
43	Implementation of new technologies	Construction method
44	Change in construction method	Construction method
45	Details, accuracy and conformity to specifications that are not appropriate	Construction method
46	Planning changes due to the results of site measurements and investigations	Construction method
47	Draft accuracy adjustment with the construction methods used	Construction method
48	Lack of availability of construction technology	Construction method
49	Poor control and testing methods of quality	Construction method
50	Damage of surrounding buildings due to project work	Construction method
51	The feasibility of the construction method	Construction method
52	Wrong test method (lab error)	Construction method
53	Lack of worker availability	Workers
54	Lack of workers capability	Workers
55	Lack of workers discipline	Workers
56	Low worker productivity	Workers
57	Lack of cohesiveness of the work team	Workers
58	Workers quarrel	Workers
59	Workers strike force	Workers
60	Decreased productivity	Workers
61	Lack of project manager skill and experience	Contractor management
62	The lack of coordination/communication	Contractor management
63	Lack of contractor skills and experience	Contractor management
64	Loss of data and documents	Contractor management
65	Incompetent and inexperienced engineer	Contractor management
66	Lack of top management support	Contractor management
67	Poor project planning and controlling	Contractor management
68	No clear authority, duties, and responsibilities (unclear task delegation)	Contractor management
69	Not administrated in project documents	Contractor management
70	Lack of supervision of subcontractors and suppliers	Operational
71	Lack of supervision of the schedule	Operational
72	Power disturbances	Operational
73	Difficulty to establish temporary facility	Operational
74	The amount of work that does not go according to plan	Operational
75	Changes to construction work due to implementation difficulty	Operational
76	Changes in supplier/contractor performance	Operational
77	Repairs due to repetitive work	Operational
78	Poor condition at locations and accessibility difficulty	Operational
79	Lack of telecommunications network provision	Operational
80	Work permission overdue	Operational

### Descriptive statistics

Descriptive statistics are used to collect, organize and process data to be presented and provide a clear picture, regarding a particular condition or event where the data is taken. Descriptive statistics are to present data clearly, in order to be taken or certain meanings. ^44^ Descriptive statistics provide an overview of the object under study through sample or population data without analyzing and making conclusions that apply to the public. ^45^ Quantitative descriptive research describes data in the form of numbers, and the size of the data includes the mean value, mode, and median. The size of the data deployment includes variance and standard deviation. ^46^ Descriptive statistical analysis determines the most influential factors on project delays, and uses mode value, which is the data that appears most often.

## Results and discussion

### Validity test

The validity test is a tool to test whether each question item truly reveals the factors or indicators that need to be investigated. ^47^ Validity testing was performed by distributing the questionnaires to 20 respondents. A validity test was performed for each variable using Pearson's product moment analysis. The variable was considered valid if the rxy value was greater than the r-table value. The r-table value obtained was 0.288, with degrees of freedom (df) associated with or an error level of 0.05, in both directions. The question had a value greater than 0.288; therefore, the questionnaire was deemed feasible and valid.

### Reliability test

A reliability test was conducted to determine whether the questionnaire was reliable, with a coefficient of ≥ 0.6. If the value was above 0.60, the questionnaire was considered reliable and feasible to use. ^48^

As shown in Table 3, a reliability coefficient of 0.958 was obtained. This shows that the coefficient of Cronbach's alpha for the variable causing the delay was greater than 0.6. Therefore, the questionnaire was deemed to be reliable.

**Table 3.** Reliability test results.

No	Factors	Cronbach alpha	Conclusion
1	80	0.958	Reliable

### Respondents and company profiles

Questionnaires were distributed to 47 respondents; their characteristics are presented in Table 4.

**Table 4.** Characteristics of respondents.

No	Group	Frequency (N = 47)	Percentage (%)
1	Company period of activity		
	0–5 years	10	21.28%
	>6–10 years	11	23.40%
	>10–15 years	8	17.02%
	>15 years	18	38.30%
2	Number of projects handled		
	1–3	5	10.64%
	>3–6	4	8.51%
	>6–10	12	25.53%
	>10	26	55.32%
3	Estimated project duration each year		
	0–6 months	41	87.23%
	>6–12 months	6	12.77%

Questionnaires were distributed to 47 respondents, and the results of distributing questionnaires on the characteristics of the respondents can be concluded based on the results of the research in Table 4. It was found that most companies, that is, 18 companies (38.30%) had over 15 years of experience in the construction sector, and the majority (26 companies, 55.32%) had handled several construction projects above 10. The majority *, i.e.*41 companies (87.23%) had estimated project durations of 0–6 months per year.

### Level of influence of project delay factors

A descriptive analysis was used to determine the level of influence of the delay risk variable. The descriptive analysis uses the mode value to determine the data that appear most often, and thus the responses that are most chosen by the respondents are obtained. The results of the levels of the factors influencing project delays are shown in Table 5.

**Table 5.** Results of descriptive statistics on the level of influence of project delay factors.

No	Description of causes	n	Mode	Level of influence
1	Increase in material prices	47	4	High
2	Delay in material delivery	47	4	High
3	Material theft	47	4	High
4	Substandard material quality	47	4	High
5	The volume and type of material is not appropriate	47	4	High
6	Damage during shipping and storage material	47	4	High
7	Limited material storage space	47	4	High
8	Supplier cannot fulfill material order	47	4	High
9	Poor material planning & management	47	4	High
10	Waste material handling	47	4	High
11	Small equipment capacity (small production)	47	4	High
12	Equipment misplacement	47	4	High
13	Delay in equipment mobilization	47	4	High
14	Incomplete equipment	47	4	High
15	Tool malfunction	47	5	Very high
16	Negligence in checking the condition of the equipment	47	4	High
17	Productivity and efficiency decreased	47	4	High
18	Additional equipment rental costs	47	4	High
19	Fuel scarcity	47	4	High
20	Difficult access for heavy equipment to be used during the execution of the project site	47	4	High
21	Poor equipment planning & management	47	4	High
22	High equipment maintenance cost	47	4	High
23	Do not understand the procedure for using equipment	47	4	High
24	Equipment not in accordance with condition	47	4	High
25	Ownership of rental equipment	47	4	High
26	Ownership of the lease-purchase equipment	47	4	High
27	Ownership of proprietary equipment	47	4	High
28	Owner does not pay on time	47	4	High
29	Cost estimation inaccuracy	47	5	Very high
30	Did not predict unexpected costs	47	4	High
31	Delay Penalty	47	4	High
32	Increased costs due to environmental safeguards	47	4	High
33	Increased work costs	47	5	Very high
34	Inefficient budgeting	47	3	Medium
35	Availability of cash	47	4	High
36	Availability of project financing sources (debtors) banks / third parties	47	4	High
37	Profit target	47	4	High
38	Unofficial charges	47	4	High
39	Financial constraints on the contractor	47	4	High
40	Investors bankruptcy	47	4	High
41	Incompatibility of the use of costs with the progress of work	47	4	High
42	Unaccurate construction method causes errors during project	47	4	High
43	Implementation of new technologies	47	5	Very high
44	Change in construction method	47	4	High
45	Details, accuracy and conformity to specifications that are not appropriate	47	5	Very high
46	Planning changes that due to the results of site measurements and investigations	47	4	High
47	Draft accuracy adjustment with the used construction methods	47	4	High
48	Lack of availability of construction technology	47	4	High
49	Poor control and testing methods of quality	47	4	High
50	Damage of surrounding buildings due to project work	47	4	High
51	The feasibility of the construction method	47	4	High
52	Wrong test method (lab error)	47	4	High
53	Lack of worker availability	47	4	High
54	Lack of workers capability	47	4	High
55	Lack of workers discipline	47	4	High
56	Low worker productivity	47	4	High
57	Lack of cohesiveness of the work team	47	4	High
58	Workers quarrel	47	5	Very high
59	Workers strike force	47	4	High
60	Decreased Productivity	47	4	High
61	Lack of project manager skill and experience	47	4	High
62	The lack of coordination / communication	47	4	High
63	Lack of contractor skills and experience	47	4	High
64	Loss of data and documents	47	4	High
65	Incompetent and inexperienced engineer	47	4	High
66	Lack of top management support	47	4	High
67	Poor project planning and controlling	47	5	Very high
68	No clear authority, duties, and responsibilities (unclear task delegation)	47	4	High
69	Not administrated in project documents	47	4	High
70	Lack of supervision of subcontractors and suppliers	47	4	High
71	Lack of supervision of the schedule	47	4	High
72	Power disturbances	47	4	High
73	Difficulty to establish temporary facility	47	4	High
74	The amount of work that does not go according to plan	47	4	High
75	Changes to construction work due to implementation difficulty	47	4	High
76	Changes in supplier / contractor performance	47	4	High
77	Repairs due to repetitive work	47	4	High
78	Poor condition at locations and accessibility difficulty	47	5	Very high
79	Lack of telecommunications network provision	47	4	High
80	Work permission overdue	47	4	High

Table 6 shows that of the 80 variables causing project delays, based on the respondents' opinions, there were eight risk factors in the very high influence category (Mode = 5), 71 factors of high influence category (Mode = 4), and one factor that belonged to the medium influence category (Mode = 3).

**Table 6.** Level influence based on number of items.

Level influence	Number of items
Very high influence	8
High influence	71
Medium influence	1
Low influence	0
Very low influence	0

**Figure 1.**
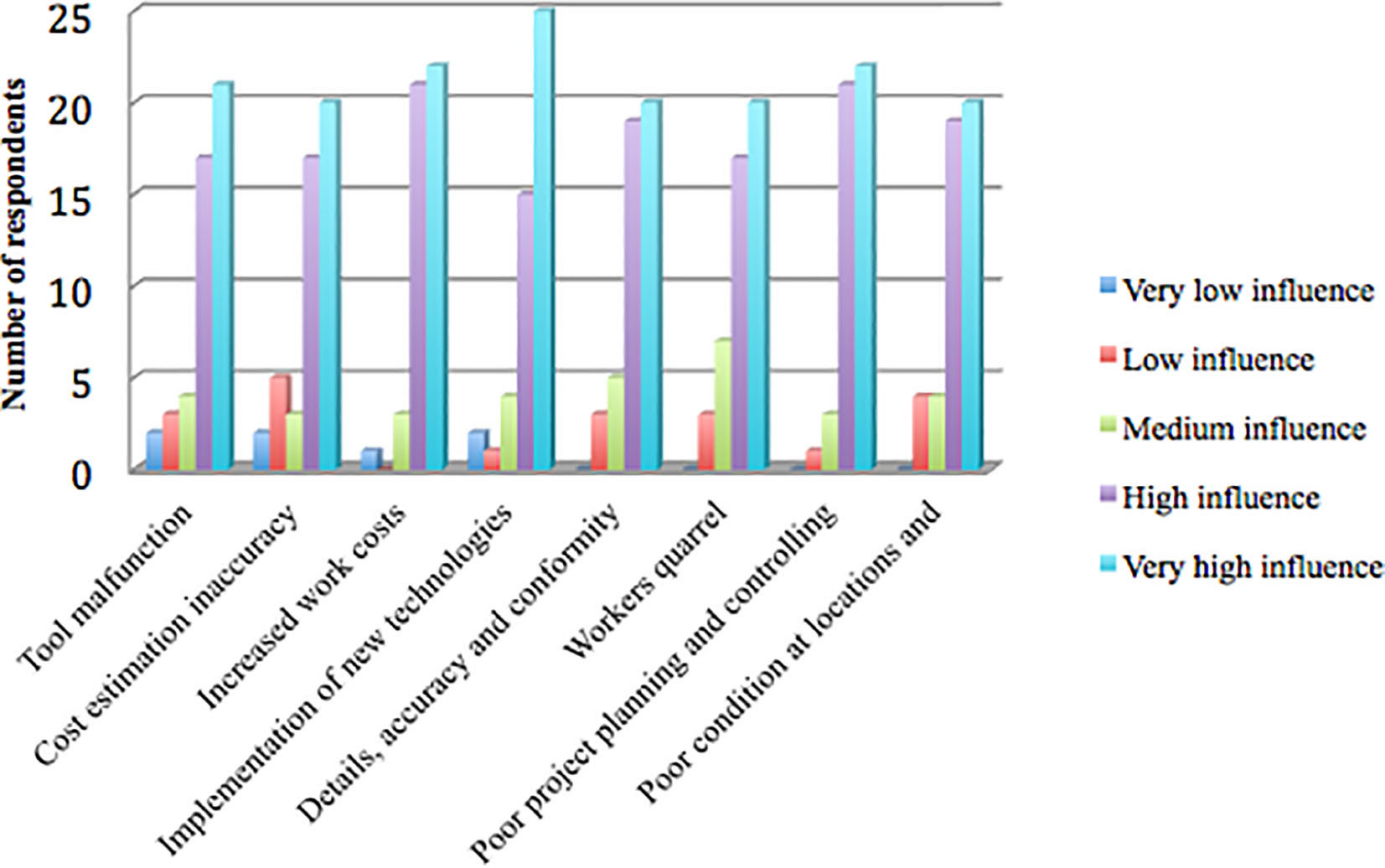
Delay factors in the very high influence category based on the respondents’ answers.

Figure 1 shows that the research results identified eight factors causing project delays that had a mode value of 5, including the very high influence category: tool malfunction, cost estimation inaccuracy, increased work costs, implementation of new technologies, details, inappropriate accuracy and conformity to specifications, workers quarrel, poor project planning and control, poor condition at locations, and accessibility difficulty.

Figure 1 shows that of the 47 respondents, 21 (44.68%) chose the very high influence category (mode =5) for the tool malfunction factor. Cost estimation inaccuracy was chosen by 20 respondents (42.55%), increased work costs by 22 respondents (46.81%), implementation of new technologies by 25 respondents (53.19%), details, accuracy, and conformity to specifications that were not appropriate by 20 respondents (42.55%), workers quarrel by 20 respondents (42.55%), poor project planning and control by 22 respondents (46.81%), poor condition at locations, and accessibility difficulty by 20 respondents (42.55%).

**Figure 2.**
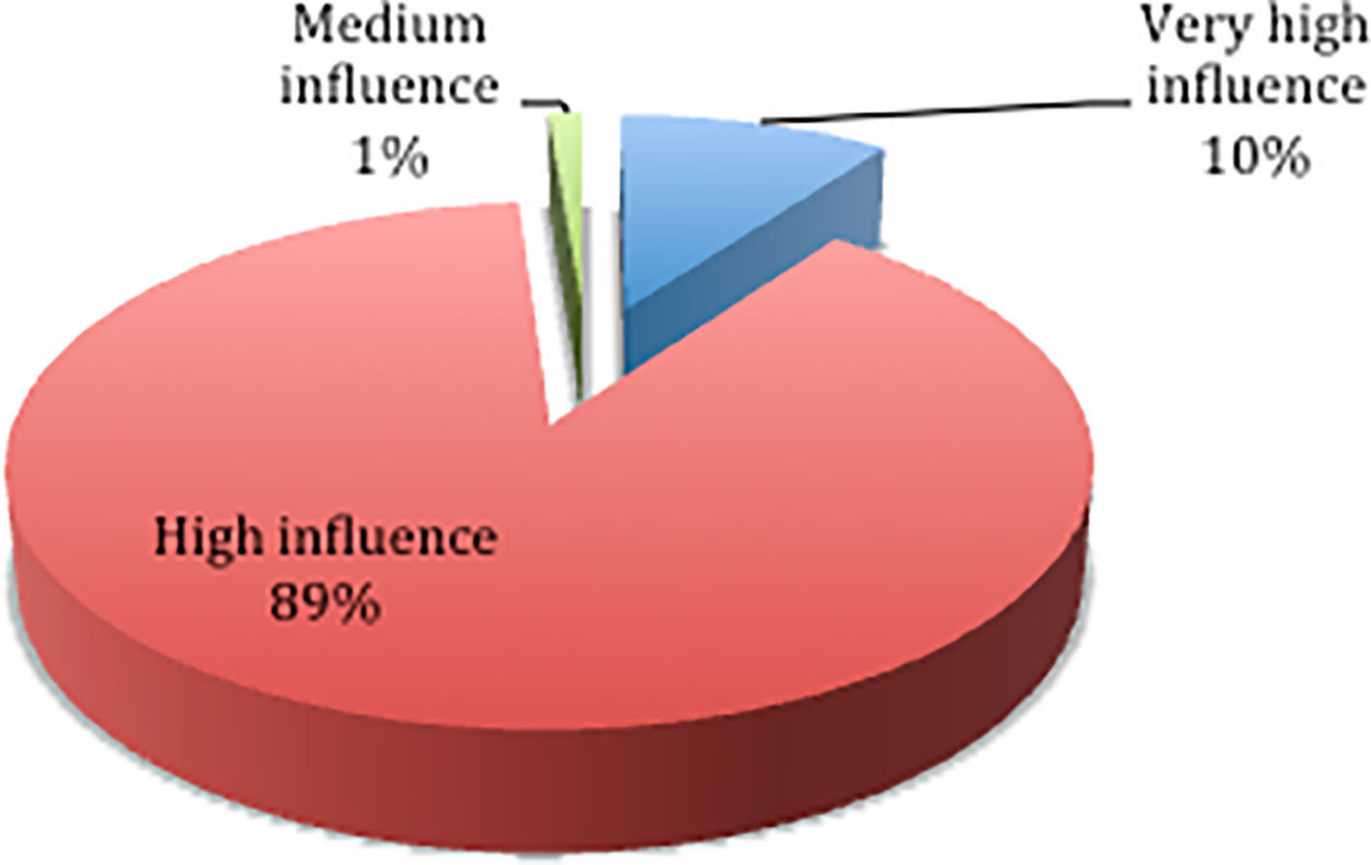
Percentage level of influence.

Based on the results of the questionnaire distribution, Figure 2 shows that 89% of respondents chose the high influence category, 10% chose the very high influence category, and 1% chose the medium influence category. The results of the descriptive statistics on the influence level of each delay factor are shown in Table 5.

*Factor 1: Tool malfunction*

The distribution of ratings for the mode value for tool malfunction was 5 (very high influence). Therefore, the results of the study indicate that the majority of respondents rated the tool malfunction indicator as having a very high influence on project delays. ^6^^,^^49^^,^^50^ Equipment damage can cause losses and endanger workers. One of the problems that often occurs is the tool’s age; the tool becomes damaged if it is too old. To avoid damage to the tool, it is best to perform routine and periodic maintenance such that the tool is more durable in operation.

*Factor 2: Cost estimation inaccuracy*

The mode value for the cost estimation inaccuracy was 5 (very high influence). Therefore, the results of the study show that the majority of respondents rated the cost estimation inaccuracy indicator as having a very high influence over project delays. Cost estimation inaccuracies can result in delays and losses. ^49^ Cost estimation is a calculation of the costs required to complete an activity or work in accordance with the requirements or contract; therefore, if the cost calculation is not appropriate, risk increases and can cause losses to the project. Therefore, accurate cost estimation is required to avoid risk. The main risk and uncertainty factor in a project is the estimated cost. ^51^

*Factor 3: Increased work costs*

The mode value for the increased work cost indicator was 5 (very high influence). Therefore, the results of the study indicate that the majority of respondents assess the indicator of increased work costs as having a very high influence on project delays. Cost overruns often occur in a project because the project implementation costs are greater than the project budget planning that has been set at the initial stage (estimated), which can cause significant losses for the project contractor. An increase in work costs is one of the causes of project delays. ^6^^,^^52^ The increase in the cost of work needs to be considered because it involves the amount of investment that must be made by the owner, where the cost overrun is vulnerable to the risk of failure. Therefore, project costs must be managed properly to minimize the possibility of cost overruns. Cost control is the final step of the project cost management process, which ensures that the use and expenditure of costs are in accordance with the planning in the form of a predetermined budget, and thus there is no increase in work costs.

*Factor 4: Implementation of new technologies*

The mode value for the implementation of new technologies was 5 (very high influence). Therefore, the results of the study indicate that the majority of respondents assessed the implementation of new technologies as having a very high influence on project delays. The development of new technological innovation and creativity is key to winning over competition and building resilience in the construction industry. ^53^ Mastery and utilization of technology is needed by construction industry players to compete globally. ^53^ The application of new and special technology that is not well known is a risk factor for project implementation because if the contractor does not know or understand new technology, it can hinder the implementation of the project, cause the project to fail or not be in accordance with the plans and losses, and can cause project delays. ^6^^,^^49^

*Factor 5: Details, accuracy and conformity to specifications that are inappropriate*

Inappropriate details, accuracy, and conformity to specifications have a mode value of 5 (very high influence). Therefore, the results of the study indicate that the majority of respondents assess the indicators of detail, accuracy, and conformity to specifications that are inappropriate as having a very high influence on project delays. However, inappropriate specifications can hinder the implementation of construction projects. ^49^

*Factor 6: Workers quarrel*

The workers’ quarrel factor had a mode value of 5 (very high influence). Therefore, the results of this study indicate that the majority of respondents assessed the indicator of workers’ quarrels as having a very high influence on project delays. Workers’ quarrels are a risk factor that can disrupt the project because if there is a fight between workers, project implementation will automatically stop and cause delays. ^6^^,^^50^ A method often used to resolve conflicts occurring between workers and in human resources on projects is a problem-solving approach, namely, discussing openly and directly using dialogue between the parties involved, identifying problems that cause conflict, seeking and collecting information on the causes of conflict, and analyzing various alternatives that are considered to be the best solution. ^54^

*Factor 7: Poor project planning and controlling*

Poor project planning and controlling factors had a mode value of 5 (very high influence). Therefore, the results of this study indicate that the majority of respondents assessed poor project planning and control as having a very high influence on project delays. Poor project planning and control are weaknesses that can lead to the possibility of a project not going as planned, and the project results are also likely not to run as expected. Therefore, poor project planning and control can result in delays and losses for the contractors. Contracting companies have a significant influence on project delays. ^55^^,^^56^ The views of clients and contractors on the causes of delays differ as they tend to blame each other for unfortunate incidents. ^14^^,^^50^^,^^57^

*Factor 8: Poor conditions at locations and accessibility difficulty*

The majority of respondents rated poor conditions and accessibility difficulty at locations as having a very high influence on project delays, where the distribution of ratings for the mode value was 5 (very high influence). Construction locations can be in poor conditions and inaccessible in the Central Aceh District, which is hilly and surrounded by mountains, making access to project sites quite difficult. Poor and difficult-to-reach project site conditions can affect project delays and potentially cause project failures ^6^^,^^58^ because of (1) a lack of initial information on field conditions, (2) contractors not conducting initial surveys, and (3) the work environment not being prepared, such as land clearing and acquisition, fresh air supply, and adequate lighting. ^59^ To avoid project delays and failures, it is expected that the contractor can collect information and conduct an initial survey regarding the condition of the project site before implementing the project such that the contractor can plan strategies for the project to run smoothly.

## Conclusions

The Indonesian government is actively engaged in construction in various sectors to create prosperity and welfare for its people. However, there are still many obstacles to working on construction projects that are not in accordance with the planned schedule. One of these obstacles is delays in construction projects. Obstacles and risks often occur during project implementation, resulting in project delays and losses. Delay in the implementation of construction projects is one of the risks that often occurs in the implementation of construction projects, especially in developing countries. Project delays for contractors can cause time and cost losses because the profits expected by the contractor are reduced, the contractor does not obtain the expected profits, or there may even be no profits at all. For project owners, delays in completing work can cause losses. Various methods have been implemented to avoid the problems that result in delays and losses. Identifying the root causes of delays is an important first step in mapping the problems that can cause project delays. The correct solution or strategy to overcome delays will be easier to obtain if the project has a map of the main factors that can cause the project to experience delays in the schedule. In this study, 80 factors causing project delays were identified, of which eight main factors were categorized as having a very high influence (=5) in causing project delays.

The findings of this study are useful for academics and construction practitioners with potentially deeper insights into the root causes of project schedule delays. The continuous expansion of knowledge and understanding of the importance (criticality) of the causes of delays will assist stakeholders in reducing the incidence of delays, lead to appropriate strategies, and can be used as comparisons or benchmarks in development planning; thus, by knowing the causes of these delays, the contractor can properly calculate these risks to avoid losses impacting the project. However, further research should be conducted with a wider study area to increase the number of respondents.

This study has limitations, namely, sampling was only conducted in the Central Aceh District, and the scope of the study is not wide enough; therefore, the results of the study cannot be generalized to a wider population. The results of this study are specific to Central Aceh Regency, and are not expandable to other regions in Indonesia. Thus, similar studies can be conducted in other districts, provinces, and cities in Indonesia, and the results of the research can be generalized to other regions. Further research is needed to increase the number of respondents such that the results are more comprehensive.

### Suggestions for future studies

Our future research will aim to determine the effects of delay factors on construction project costs using the ordinal logistic regression method. Future research will be conducted to determine the delay factors that have a significant effect on construction project costs. These delay factors are expected to serve as a reference for contractor companies carrying out construction projects such that they can avoid construction project losses.

## Data availability

### Underlying data

Zenodo: Raw data for the study of the f1000 manuscript entitled: Critical Delay Factors for Construction Project in Central Aceh District, Indonesia

This project contains the following underlying data:

- Raw Data(1).xlsx

### Extended data

Zenodo: Raw data for the study of the f1000 manuscript entitled: Critical Delay Factors for Construction Project in Central Aceh District, Indonesia

This project contains the following extended data:

- Questionnaire1.xlsx

Data are available under the terms of the Creative Commons Attribution 4.0 International license (CC-BY 4.0).

## References

[1] Syam F : Community Leader of Central Aceh Regency: If the Multiyear Project Is Canceled, We Can Ask for Separation. *Aceh Journal National Network.* 2020. (accessed Feb. 07, 2021). Reference Source

[2] Qazi A Quigley J Dickson A : Project Complexity and Risk Management (ProCRiM): Towards modelling project complexity driven risk paths in construction projects. *Int. J. Proj. Manag.* 2016;34:1183–1198. 10.1016/J.IJPROMAN.2016.05.008

[3] Gray CF Larson EW : *Project Management: The Managerial Process.* Singapore: McGraw-Hill;2000.

[4] Rauzana A : The effect of the risk factors on the performance of contractors in Banda Aceh, indonesia. *ARPN J. Eng. Appl. Sci.* 2016;11(15):9496–9502.

[5] Alsuliman JA : Causes of delay in Saudi public construction projects. *Alex. Eng. J.* 2019;58(2):801–808. 10.1016/j.aej.2019.07.002

[6] Aziz RF Abdel-Hakam AA : Exploring delay causes of road construction projects in Egypt. *Alex. Eng. J.* 2016;55(2):1515–1539. 10.1016/j.aej.2016.03.006

[7] Mahdi I Soliman E : Significant and top ranked delay factors in Arabic Gulf countries. *Int. J. Constr. Manag.* 2019;21(14):167–180. 10.1080/15623599.2018.1512029

[8] Mpofu B Godfrey E Moobela OC : Profiling causative factors leading to construction project delays in the United Arab Emirates. *Eng. Constr. Archit. Manag.* 2017;24(2):346–376. 10.1108/ECAM-05-2015-0072

[9] Al Amri T Marey-Pérez M : Towards a sustainable construction industry: Delays and cost overrun causes in construction projects of Oman. *J. Proj. Manag.* 2020;5:87–102. 10.5267/j.jpm.2020.1.001

[10] Rumimper R : Risk Analysis on Housing Construction Projects in Minahasa-North Regency. *Sci. J. Media Eng.* 2015;5(2):381–389.

[11] Yap JB Goay PL Woon YB : Revisiting critical delay factors for construction: Analysing projects in Malaysia. *Alex. Eng. J.* 2021;60(1):1717–1729. 10.1016/j.aej.2020.11.021

[12] Alwi S Hampson K : Identifying the important causes of delays in building construction projects. *Proceedings Ninth EastAsia-Pacific Conference on Structural Engineering and Construction, Bali, Indonesia.* 2003; pp.1–6.

[13] Abu Salem ZT Suleiman A : Risk factors causing time delay in the Jordanian construction sector. *Int. J. Eng. Res. Technol.* 2020;13(2):307–315. 10.37624/ijert/13.2.2020.307-315

[14] Yap JBH Skitmore M : Investigating design changes in Malaysian building projects. *Archit. Eng. Des. Manag.* 2018;14(3):218–238. 10.1080/17452007.2017.1384714

[15] Zarei B Sharifi H Chaghouee Y : Delay causes analysis in complex construction projects: a Semantic Network Analysis approach. *Prod. Plan. Control.* 2018;29(1):29–40. 10.1080/09537287.2017.1376257

[16] Shahsavand P Marefat A Parchamijalal M : Causes of delays in construction industry and comparative delay analysis techniques with SCL protocol. *Eng. Constr. Archit. Manag.* 2018;25(4):497–533. 10.1108/ECAM-10-2016-0220

[17] Gbahabo PT Ajuwon OS : Effects of project cost overruns and schedule delays in Sub- Saharan Africa. *Eur. J. Interdiscip. Stud.* 2017;3(2):46–58. 10.26417/EJIS.V7I2.P46-59

[18] Sambasivan M Deepak TJ Salim AN : Analysis of delays in Tanzanian construction industry: Transaction cost economics (TCE) and structural equation modelling (SEM) approach. *Eng. Constr. Archit. Manag.* 2017;24(2):308–325. 10.1108/ECAM-09-2015-0145

[19] Aibinu AA Jagboro GO : The effects of construction delays on project delivery in Nigerian construction industry. *Int. J. Proj. Manag.* 2002;20(8):593–599. 10.1016/S0263-7863(02)00028-5

[20] Wu Y Zhou J : Risk assessment of urban rooftop distributed PV in energy performance contracting (EPC) projects: An extended HFLTS-DEMATEL fuzzy synthetic evaluation analysis. *Sustain. Cities Soc.* 2019;47(1):101524. 10.1016/j.scs.2019.101524

[21] PMI: *A guide to the project management body of knowledge (PMBOK guide).* 6th ed. USA: Newton Square, USA: Project Management Institute;2017.

[22] Qazi A Dikmen I : From risk matrices to risk networks in construction projects. *IEEE Trans. Eng. Manag.* 2019;68(12):1449–1460. 10.1109/TEM.2019.2907787

[23] Ansah RH Sorooshian S : Effect of lean tools to control external environment risks of construction projects. *Sustain. Cities Soc.* 2017;32:348–356. 10.1016/J.SCS.2017.03.027

[24] Hwang B Ngo J Her PWY : Integrated Digital Delivery: Implementation status and project performance in the Singapore construction industry. *J. Clean. Prod.* 2020;262:121396. 10.1016/j.jclepro.2020.121396

[25] Eskander RFA : Risk assessment influencing factors for Arabian construction projects using analytic hierarchy process. *Alex. Eng. J.* 2018;57(4):4207–4218. 10.1016/j.aej.2018.10.018

[26] Rauzana A : Identification and Assessment of Risk Factors Affecting Construction Projects in North Aceh, Indonesia. *IOSR J. Bus. Manag.* 2016;18(09):72–77. 10.9790/487x-1809047277

[27] Rauzana A : The Influence of Uncertainty Variables on Cost Estimation Lesson Learned From Construction Industry in Indonesia. *Aust. J. Basic Appl. Sci.* 2015;9(April):380–385.

[28] Leu SS Chen AT Yang CH : A GA-based optimal model for construction time-cost trade-off. *Int. J. Proj. Manag.* 2001;19(1):47–58. 10.1016/S0263-7863(99)00035-6

[29] PMI: *Construction Extension to the PMBOK ^®^ Guide.* Project Management Institute, Inc.;2016.

[30] Stumpf GR : Schedule delay analysis. *Cost Eng. Arbor Then Morgant.* 2000;42(7):32–32.

[31] Assaf SA Al-Hejji S : Causes of delay in large construction projects. *Int. J. Proj. Manag.* 2006;24(4):349–357. 10.1016/j.ijproman.2005.11.010

[32] Central Aceh District Government: *Integrated Plan And Medium-Term Infrastructure Investment Plan, Central Aceh District, 2016-2020.* Central Aceh District;2020.

[33] Kasidi: *Risk Management.* Bogor: Ghalia Indonesia;2010.

[34] Rauzana A Dharma W : The knowledge and awareness of occupational health and safety requirements among civil engineering students in an Indonesian university.vol.23(no.3): pp.210–215.2021.

[35] Suárez M García-Romero E Baz A : Smectites: The key to the cost overruns in the construction of the third set of locks of the Panama Canal. *Eng. Geol.* 2021;284:106036. 10.1016/j.enggeo.2021.106036

[36] Nasution S : *Research Methods (Scientific Research).* Jakarta: Bumi Aksara; 6th ed. 2003.

[37] Le-Hoai L Lee YD Lee JY : Delay and cost overruns in Vietnam large construction projects: A comparison with other selected countries. *KSCE J. Civ. Eng.* 2008;12(6):367–377. 10.1007/s12205-008-0367-7

[38] Bagaya O Song J : Empirical study of factors influencing schedule delays of public construction projects in Burkina Faso. *J. Manag. Eng.* 2016;32(5):05016014. 10.1061/(ASCE)ME.1943-5479.0000443

[39] Sugiyono: *Combined Research Methods (Mixed Methods).* Bandung: Alfabeta;2013.

[40] Riduwan and Sunarto: *Introduction to Statistics for Research: Education, Social, Communication, Economics, and Business.* Bandung: Alfabeta;2014.

[41] Vagias WM : *Likert-type scale response anchors. Clemson International Institute for Tourism & Research Development.* Department of Parks, Recreation and Tourism Management, Clemson University;2006.

[42] Sugiyono: *Combination Research Methods.* Bandung: Alfabeta;2015.

[43] Rauzana A Dharma W : Causes of delays in construction projects in the Province of Aceh, Indonesia. *PLoS One.* 2022;17(1):e0263337. 10.1371/journal.pone.0263337 35089971 PMC8797244

[44] Hasan I : *Research Data Analysis with Statistics.* Jakarta: PT Bumi Aksara;2004.

[45] Jaya I : *Application of Statistics for Educational Research.* jakarta, Indonesia: Prenadamedia Group;2019.

[46] Kaur P Stoltzfus J Yellapu V : Descriptive statistics. *Int. J. Acad. Med.* 2018;4(1):60–63. 10.4103/IJAM.IJAM_7_18

[47] Arikunto S : *Research Management.* Jakarta: Rineka Cipta;2013.

[48] Sekaran U : *Research methods for business: A skill building approach.* 4th ed. John Wiley & Sons;2006.

[49] Sangari F Tjakra J : Risk Analysis of Housing Construction Projects in Manado City. *Sci. J. Media Eng.* 2011;1(1):29–37.

[50] Aziz RF : Ranking of delay factors in construction projects after Egyptian revolution. *Alex. Eng. J.* 2013;52(3):387–406. 10.1016/j.aej.2013.03.002

[51] Aidil M Rauzana A Muhammad N : A Study on Drinking Water Distribution Project in Banda Aceh. *J. Phys. Conf. Ser.* 2021;1933(1):012091. 10.1088/1742-6596/1933/1/012091

[52] Rodrigues-da-Silva LH Crispim JA : The project risk management process, a preliminary study. *HCIST J.* 2014;16:943–949. 10.1016/j.protcy.2014.10.047

[53] Kusumawanti R : *Construction Industry Needs New Technology.* Portonews;2019. (accessed Jun. 07, 2021). Reference Source

[54] Susila H : Conflict Handling Methods In The Implementation Of Building Construction Projects In Surakarta. *J. Civ. Archit. Eng.* 2012;12(16):6–10.

[55] Mydin MAO Sani NMM Taib NM : Imperative causes of delays in construction projects from developers’ outlook. 2014. 10.1051/matecconf/20141006005

[56] Rao PB Camron CJ : Causes of delays in construction projects: A case study. *Int. J. Curr. Res.* 2014;6(6):7219–7222.

[57] Al-Kharashi A Skitmore M : Causes of delays in Saudi Arabian public sector construction projects. *Constr. Manag. Econ.* 2009;27(1):3–23. 10.1080/01446190802541457

[58] Muka I : Risk Analysis in the Sulawesi Denpasar Road Basement Parking Development Project. *J. Civ. Eng. Commun. Media.* 2012;19(2):155–165. 10.14710/mkts.v19i2.8425

[59] Price D Andy K : Causes Leading to Poor site Coordination in Building Projects. *Organ. Technol. Manag. Constr. - An Int. J.* 2010;2(2):167–172.

