# Critical delay factors for construction projects in Central Aceh District, Indonesia

**DOI:** 10.12688/f1000research.110024.3

**Published:** 2022-09-29

**Authors:** Anita Rauzana, Aghnia Zahrah, Wira Dharma

**Affiliations:** 1 Department of Civil Engineering, Universitas Syiah Kuala, Banda Aceh, 23111, Indonesia; 2 Department of Architecture and Planning, Universitas Syiah Kuala, Banda Aceh, 23111, Indonesia; 3 Faculty of Mathematics and Natural Sciences, Universitas Syiah Kuala, Banda Aceh, 23111, Indonesia

**Keywords:** delay factors, project management, risk management, construction industry, schedule delays

## Abstract

**Background:** Construction development in Indonesia is growing rapidly, especially in Central Aceh District. Construction projects often experience risks, so that risk events can have a serious impact on the viability of the project. Project delays can result in cost overruns and project losses. Therefore, it is necessary to identify the factors causing project delays.The purpose of this study was to (1) identify the risk factors that cause delays in construction projects and (2) determine those particular risk factors that have a greater influence on construction projects. The location of this research was Central Aceh District.

**Methods:** The data in this study were primary data in the form of a questionnaire and secondary data obtained from the literature related to this particular type of research. Questionnaires were distributed to respondents, namely contractor companies located in the Central Aceh District. The questionnaires were distributed to determine respondents' opinions about the level of influence of risk factors causing project delays. We used a validity test, reliability test, and descriptive analysis for data processing.

**Results:** Based on the results of the study from 47 respondents, the “very high influence” category (Mode=5) for the tool malfunction factor, cost estimation inaccuracy, increased work costs, implementation of new technologies, details, accuracy and conformity to specifications that are not appropriate, worker quarrels, poor project planning and management, poor condition at locations and accessibility difficulty.

**Conclusions:** Of the 80 risk factors that caused project delays, eight risk factors were found to have a very high influence on the implementation of construction projects in Central Aceh District.

**Practical implications:** The results of this study provide knowledge to contractor companies about the delay factors that have the most influence on project implementation so that they are expected to be able to manage risks to avoid losses.

## Introduction

The Central Aceh Regency is one of the regions in Aceh Province, Indonesia, experiencing rapid growth. This can be observed in the number of construction projects currently underway. ^1^ The implementation of construction projects in the Central Aceh Regency often experiences failures and delays, ^1^ which can cause project losses. The project has limited resources, namely labor, money, materials, equipment, and project resources must be used effectively and efficiently to avoid losses. A complex project can cause high-risk and uncertain events that can cause delays and cost overruns on projects, ^2^ thus allowing for uncertainty in the implementation process, which leads to various types of risks that ultimately cause losses to the parties involved in the construction project and affect the achievement of the desired goal. Risk is a condition in which there is a possibility of gain/loss, ^3^ with losses, such as cost losses, injuries, and delays caused by uncertainty during project implementation. One of the most influential risk factors is changing the order. ^4^ Delays in the implementation of projects are among the risks that often occur in the implementation of construction projects, especially in developing countries. ^5^ ^–^ ^8^ Project delays and cost overruns can harm projects. ^9^

The purpose of this study was to (1) identify the risk factors that cause delays in construction projects and (2) determine those particular risk factors that have a greater influence on construction projects. The location of this research was Central Aceh District, Indonesia.

An increase in fuel prices can cause cost increases, losses, and delays in construction projects. ^6^ ^,^ ^10^ There are five causes for a project loss: (1) improper planning and scheduling, (2) many changes to orders by clients, (3) incompetent site management and supervision, (4) inexperienced subcontractors, and (5) poor contractor finances. ^11^ Experienced contractors can accelerate a project schedule. ^12^ The most influential risk factor for projects in Jordan (the Middle East) is poor soil/site conditions in construction projects. ^13^

Previous research has shown that delays in project implementation can lead to cost overruns. ^14^ Delays affect planning and control, ^15^ especially during project implementation. ^16^ Project delays can lead to losses, legal problems, and contract termination. ^17^ ^,^ ^18^ The contractor suffers losses owing to cost overruns. For example, in Nigeria, cost overruns and delays are frequent factors affecting projects. ^19^ It is important to apply risk management to avoid project failure because construction projects are complex and involve many risks. ^20^ Project risk is defined as an unforeseen event or situation that can harm a project. ^21^ Risk management is an important process for achieving project objectives. ^22^ ^,^ ^23^ Identifying, assessing, and managing construction project risks is indispensable for risk management. A successful project is time- and cost-effective, and has good construction quality. ^24^ Managing risk is an important mechanism in the construction sector, which is performed to obtain project objectives in the form of cost, time, safety, and good quality ^25^; the most influential risk factor is material. ^26^

Development projects globally often involve considerable risk. Inflation causes delays and losses. ^27^ Risks can affect the time, cost, quality, and performance of a construction project. ^28^ Time risk affects project costs. Project risk management aims to increase profits and reduce losses. ^29^

For construction projects, overtime or delays are common during project implementation. Time delays can be described as events or interruptions that result in a project not being completed within the time specified in the contract. Defining delays as actions or activities that increase the time required by the contractor to conduct the project is referred to as time contingency. ^30^

Only 30% of Saudi Arabian construction projects implemented require an average additional time of approximately 10–30%, where there are nine main groups of risk factors causing delays: costs, resources, contracts, schedule, government relations, personnel, planning, equipment, and environmental factors. Funding delay is the most important delay factor. ^31^

The risk of delays often occur in construction projects in various countries based on previous studies, it is necessary to conduct a similar study in Central Aceh District, Indonesia, given that construction projects in Central Aceh District often experience delays. ^1^ The causes of project delays in the Central Aceh District are land acquisition constraints and financial problems. ^32^ In project implementation, the contractor company does not know the risk of project delays. Therefore, to avoid losses and delays in construction projects, research is needed to identify and analyze the factors causing delays in construction projects, particularly in Central Aceh District, given the complex conditions of the district, including socio-cultural diversity, high inflation rates, low public education, frequent disasters, community economy weakness, geographic location, social and political conflicts, and economic crises. ^33^

Risk identification is conducted by collecting all information related to activities and analyzing it to find every possible risk that could result in a loss. Risk identification can be performed using several techniques. ^34^ Identifying risks in a project consists in compiling (1) a list of risks that can cause losses, (2) a list of potential losses, and in this checklist compiling (3) a list of losses and (4) a ranking of losses occurring, and then (5) classifying losses. Project delays also occur owing to work accidents. ^35^ The type of soil and rock at the project site is one of the main risk factors for project delay. ^36^

## Literature review

### Causes of project delays

In a construction project, many things may happen which can increase the time of activity or delay the completion of a project as a whole. Some of the most common causes include changes in field conditions, changes in design or specifications, weather changes, and unavailability of manpower, materials, or equipment. ^37^ Delays in project implementation can cause losses and one of the risks that often occurs in the implementation of construction projects. ^5^ ^–^ ^7^

Construction project delays are caused by errors in estimating the costs and time required to complete the project, ^38^ or various possibilities, for example, due to improper management, material problems, labor, equipment, finances, and an unsupportive environment so that delays occur, ^11^ ^,^ ^39^ and may result in project delays. Project delays for the contractor will experience a cost loss, because the profits expected by the contractor will be reduced, or even no profit will be made at all. For the Owner, delays in the completion of project work will cause losses to the operating time of the project results, so the use of project development will be delayed. ^14^ Factors causing project delays based on previous studies can be seen in Table 1.

**Table 1.** Factors causing project delays based on previous studies.

No	Description of causes	Category	Previous literature
1	Increase in material prices	Material	^6^ ^,^ ^27^ ^,^ ^37^ ^,^ ^38^ ^,^ ^40^ ^,^ ^41^
2	Delay in material delivery	Material	^6^ ^,^ ^27^ ^,^ ^37^ ^,^ ^38^ ^,^ ^40^ ^,^ ^41^
3	Material theft	Material	^6^ ^,^ ^27^ ^,^ ^37^ ^,^ ^38^ ^,^ ^40^ ^,^ ^41^
4	Substandard material quality	Material	^6^ ^,^ ^27^ ^,^ ^37^ ^,^ ^38^ ^,^ ^40^ ^–^ ^42^
5	The volume and type of material is not appropriate	Material	^6^ ^,^ ^27^ ^,^ ^37^ ^,^ ^38^ ^,^ ^40^ ^,^ ^41^
6	Damage during shipping and storage material	Material	^6^ ^,^ ^27^ ^,^ ^37^ ^,^ ^38^ ^,^ ^40^ ^–^ ^42^
7	Limited material storage space	Material	^6^ ^,^ ^27^ ^,^ ^37^ ^,^ ^38^ ^,^ ^40^ ^,^ ^41^
8	Supplier cannot fulfil material order	Material	^6^ ^,^ ^27^ ^,^ ^37^ ^,^ ^38^ ^,^ ^40^ ^–^ ^42^
9	Poor material planning & management	Material	^6^ ^,^ ^27^ ^,^ ^37^ ^,^ ^38^ ^,^ ^40^ ^,^ ^41^
10	Waste material handling	Material	^6^ ^,^ ^27^ ^,^ ^37^ ^,^ ^38^ ^,^ ^40^ ^,^ ^41^
11	Small equipment capacity (small production)	Equipment	^6^ ^,^ ^11^ ^,^ ^43^
12	Equipment misplacement	Equipment	^6^ ^,^ ^40^ ^,^ ^41^ ^,^ ^43^
13	Delay in equipment mobilization	Equipment	^6^ ^,^ ^37^ ^,^ ^40^ ^,^ ^41^
14	Incomplete equipment	Equipment	^6^ ^,^ ^11^ ^,^ ^43^
15	Tool malfunction	Equipment	^6^ ^,^ ^11^ ^,^ ^43^
16	Negligence in checking the condition of the equipment	Equipment	^6^ ^,^ ^37^ ^,^ ^40^ ^,^ ^41^
17	Productivity and efficiency decreased	Equipment	^6^ ^,^ ^37^ ^,^ ^40^ ^,^ ^41^
18	Additional equipment rental costs	Equipment	^6^ ^,^ ^37^ ^,^ ^40^ ^,^ ^41^
19	Fuel scarcity	Equipment	^6^ ^,^ ^37^ ^,^ ^40^ ^,^ ^41^ ^,^ ^44^
20	Difficult access for heavy equipment to be used during the execution of the project site	Equipment	^6^ ^,^ ^37^ ^,^ ^40^ ^,^ ^41^ ^,^ ^44^
21	Poor equipment planning & management	Equipment	^6^ ^,^ ^37^ ^,^ ^40^ ^,^ ^41^ ^,^ ^44^ ^,^ ^45^
22	High equipment maintenance cost	Equipment	^6^ ^,^ ^37^ ^,^ ^40^ ^,^ ^41^ ^,^ ^44^ ^,^ ^46^
23	Do not understand the procedure for using equipment	Equipment	^6^ ^,^ ^37^ ^,^ ^40^ ^,^ ^41^ ^,^ ^44^ ^,^ ^46^
24	Equipment not in accordance with condition	Equipment	^6^ ^,^ ^11^ ^,^ ^43^
25	Ownership of rental equipment	Equipment	^6^ ^,^ ^37^ ^,^ ^40^ ^,^ ^41^ ^,^ ^44^ ^,^ ^46^
26	Ownership of the lease-purchase equipment	Equipment	^6^ ^,^ ^11^ ^,^ ^43^
27	Ownership of proprietary equipment	Equipment	^6^ ^,^ ^37^ ^,^ ^40^ ^,^ ^41^ ^,^ ^44^ ^,^ ^46^
28	Owner does not pay on time	Financial	^6^ ^,^ ^10^ ^,^ ^46^
29	Cost estimation inaccuracy	Financial	^6^ ^,^ ^10^ ^,^ ^38^ ^,^ ^40^
30	Did not predict unexpected costs	Financial	^6^ ^,^ ^10^ ^,^ ^38^ ^,^ ^43^
31	Delay penalty	Financial	^6^ ^,^ ^40^ ^,^ ^43^ ^,^ ^46^
32	Increased costs due to environmental safeguards	Financial	^6^ ^,^ ^46^
33	Increased work costs	Financial	^6^ ^,^ ^43^
34	Inefficient budgeting	Financial	^6^ ^,^ ^19^ ^,^ ^38^
35	Availability of cash	Financial	^6^ ^,^ ^19^ ^,^ ^46^
36	Availability of project financing sources (debtors) banks/third parties	Financial	^6^ ^,^ ^19^ ^,^ ^46^
37	Profit target	Financial	^6^ ^,^ ^38^ ^,^ ^46^
38	Unofficial charges	Financial	^6^ ^,^ ^46^
39	Financial constraints on the contractor	Financial	^6^ ^,^ ^46^
40	Investors bankruptcy	Financial	^6^ ^,^ ^46^
41	Incompatibility of the use of costs with the progress of work	Financial	^6^ ^,^ ^46^
42	Inaccurate construction method causes errors during project	Construction method	^40^ ^,^ ^46^
43	Implementation of new technologies	Construction method	^37^ ^,^ ^40^ ^,^ ^46^
44	Change in construction method	Construction method	^4^ ^,^ ^37^ ^,^ ^40^ ^,^ ^46^
45	Details, accuracy and conformity to specifications that are not appropriate	Construction method	^11^ ^,^ ^40^ ^,^ ^46^ ^,^ ^47^
46	Planning changes due to the results of site measurements and investigations	Construction method	^11^ ^,^ ^40^ ^,^ ^46^ ^,^ ^47^
47	Draft accuracy adjustment with the construction methods used	Construction method	^11^ ^,^ ^40^ ^,^ ^46^ ^,^ ^47^
48	Lack of availability of construction technology	Construction method	^11^ ^,^ ^40^ ^,^ ^46^ ^,^ ^47^
49	Poor control and testing methods of quality	Construction method	^11^ ^,^ ^40^ ^,^ ^46^ ^–^ ^48^
50	Damage of surrounding buildings due to project work	Construction method	^47^ ^,^ ^48^
51	The feasibility of the construction method	Construction method	^11^ ^,^ ^40^ ^,^ ^46^ ^,^ ^47^
52	Wrong test method (lab error)	Construction method	^11^ ^,^ ^40^ ^,^ ^46^ ^,^ ^47^
53	Lack of worker availability	Workers	^27^ ^,^ ^40^
54	Lack of workers capability	Workers	^27^ ^,^ ^40^
55	Lack of workers discipline	Workers	^27^ ^,^ ^40^
56	Low worker productivity	Workers	^27^ ^,^ ^40^
57	Lack of cohesiveness of the work team	Workers	^27^ ^,^ ^40^
58	Workers quarrel	Workers	^27^ ^,^ ^44^
59	Workers strike force	Workers	^27^ ^,^ ^44^
60	Decreased productivity	Workers	^27^ ^,^ ^44^
61	Lack of project manager skill and experience	Contractor management	^27^ ^,^ ^49^
62	The lack of coordination/communication	Contractor management	^27^ ^,^ ^40^ ^,^ ^49^ ^,^ ^50^
63	Lack of contractor skills and experience	Contractor management	^27^ ^,^ ^40^ ^,^ ^49^
64	Loss of data and documents	Contractor management	^27^ ^,^ ^40^ ^,^ ^49^ ^,^ ^50^
65	Incompetent and inexperienced engineer	Contractor management	^37^ ^,^ ^40^ ^,^ ^50^
66	Lack of top management support	Contractor management	^27^ ^,^ ^40^ ^,^ ^49^ ^,^ ^50^
67	Poor project planning and controlling	Contractor management	^27^ ^,^ ^49^ ^,^ ^50^
68	No clear authority, duties, and responsibilities (unclear task delegation)	Contractor management	^37^ ^,^ ^38^ ^,^ ^50^
69	Not administrated in project documents	Contractor management	^27^ ^,^ ^37^ ^,^ ^50^
70	Lack of supervision of subcontractors and suppliers	Operational	^11^ ^,^ ^27^ ^,^ ^40^ ^,^ ^42^
71	Lack of supervision of the schedule	Operational	^11^ ^,^ ^27^ ^,^ ^40^ ^,^ ^42^
72	Power disturbances	Operational	^11^ ^,^ ^27^ ^,^ ^40^ ^,^ ^42^
73	Difficulty to establish temporary facility	Operational	^11^ ^,^ ^27^ ^,^ ^40^ ^,^ ^42^
74	The amount of work that does not go according to plan	Operational	^11^ ^,^ ^27^ ^,^ ^40^ ^,^ ^42^
75	Changes to construction work due to implementation difficulty	Operational	^11^ ^,^ ^27^ ^,^ ^40^ ^,^ ^42^
76	Changes in supplier/contractor performance	Operational	^27^ ^,^ ^37^ ^,^ ^48^ ^–^ ^50^
77	Repairs due to repetitive work	Operational	^11^ ^,^ ^27^ ^,^ ^40^ ^,^ ^42^
78	Poor condition at locations and accessibility difficulty	Operational	^11^ ^,^ ^27^ ^,^ ^40^ ^,^ ^42^
79	Lack of telecommunications network provision	Operational	^11^ ^,^ ^27^ ^,^ ^40^ ^,^ ^42^
80	Work permission overdue	Operational	^11^ ^,^ ^27^ ^,^ ^40^ ^,^ ^42^

### Effects of project delays on stakeholders

A construction project is a series of activities that are limited by resources and time, which aim to achieve construction results with good quality standards. The achievement of a good construction project must be supported by proper planning, and optimal resources. ^27^

Stakeholder involvement is a project success factor that has a positive effect on minimizing cost overruns and schedule delays. Stakeholders who are directly and indirectly involved in construction projects are required to have competitive services through creative, innovative, and efficient efforts so that all understand the needs and expectations of project quality at present and in the future. Every stage of the project can not be separated from various risks and uncertainties.

Construction risk in general is an event that affects project objectives, time costs, and quality. ^3^ With various trends that occur in the field, it appears that there is a gap between the conditions in the field and the ideal situation that should occur. Construction projects do not always run smoothly and often face problems related to the influence of stakeholders, which can cause cost increases and delays. ^6^ ^,^ ^43^ Stakeholders are not optimal in supporting project success or even hindering project objectives. Conditions should be created in which stakeholders are expected to support the success of the project. Project failures are often caused by late payments, labor-related problems, subcontractors/main contractors, and insufficient contingency costs. ^51^

### Project risk factors

Risk identification is the cornerstone of an accident prevention or risk control programme. Without knowing the hazard, the risk cannot be determined so that risk prevention and control efforts cannot be carried out. Risk identification provides various benefits, including: ^52^

1. Reducing the chance of accidents, because the identification of hazards is related to the factors that cause accidents. By identifying risks, various sources of danger that trigger accidents can be identified and then eliminated so that the possibility of accidents can be reduced.
2. To provide an understanding for all parties (management workers and other related parties) regarding the potential dangers of the company’s activities so as to increase vigilance in running the company.
3. As a basis as well as input for determining appropriate and effective prevention and security strategies.
4. Provide documented information regarding the sources of hazards within the company to all parties, especially stakeholders.

The way to identify risks in a project is to compile (1) a list of risks that can cause losses, (2) a list of potential losses, and in this checklist compile (3) a list of losses and (4) a ranking of losses occuring, and then (5) make a classification of losses. During project implementation, there are many risk and uncertainty variables that dynamically affect the duration of activities, as well as costs. ^28^ Many of the uncertainties associated with international construction arise from differences in culture, economic conditions, specifications or standards, legal frameworks, and levels of productivity. ^53^ The history of the construction industry is replete with projects completed at significant cost overruns. ^54^ One of the main risk factors for project delays is the type of soil and rock at the project site. ^35^

Cost overruns in construction projects are not a new problem in the construction industry, and they are recognised as a global problem. Cost overruns have occurred at an average rate of 28% over a period of 70 years. ^55^ Cost estimation is very important in the early stages of construction project implementation, and becomes a very important event with which to consider the beginning of project planning. ^56^ Therefore, efficient cost estimation is very important to avoid project loss. Projects with too high a cost can increase construction costs, increase pressure on investors, reduce potential investor decision-making, and create huge national financial losses. ^57^ ^,^ ^58^ Project success has three indicators, namely, efficient cost, effective time, and good quality. Unfortunately, many construction projects experience cost deviations from the initial set budget, and this happens because of the effects of risk and uncertainty on project implementation. ^45^

Mahamid notes 43 causes of delays in road construction projects in Palestine. ^59^ The study of these factors shows that there are eight variables in the red zone of the risk matrix. It was found that the causes of delay risk were financial problems, and inexperienced contractors. ^60^ The most significant risk factor is the contractors behaviour and (in) experience. ^61^ The main causes of delays are change orders, the owners financial constraints, and the owners lack of interest. ^62^

### The case study district

Central Aceh district is an area that is part of the province of Nanggroe Aceh Darussalam, Indonesia, whose capital is Takengon. Takengon is an area inhabited by local ethnic and ethnic immigrants, this area is generally inhabited by most of the Gayo tribe as the majority group. Central Aceh is one of the districts located in the middle of Aceh Province, Indonesia. Its area is 431,839 Ha or equivalent to 4,318.39 km ^2^, directly adjacent to Bener Meriah and Bireuen Regencies in the north, Gayo Lues Regency in the south, Nagan Raya and Pidie Regencies in the west, and East Aceh Regency in the east.

Central Aceh Regency is administratively divided into 14 sub-districts consisting of 269 definitive villages and 27 preparatory villages. In the first quarter of 2011, the population reached 202,114 people with an average density of 47 people/km ^2^. The composition of the population is Gayo ethnicity ± 60%, Javanese 30%, Aceh Coastal 5%, and the rest are other ethnic groups such as Batak, Padang, and Chinese.

The Central Aceh Regency is one of the regions in Aceh Province, Indonesia, experiencing rapid growth. This can be observed in the number of construction projects currently underway. ^1^ The implementation of construction projects in the Central Aceh Regency often experiences failures and delays. ^32^

## Methods

### Questionnaire design

The primary data in this study was questionnaire data; the questionnaire was distributed to 47 respondents and contained 80 questions about project delays. Secondary data were obtained from studies in the literature such as journals, books, and other literature related to this research, as well as data about contractor companies obtained from the National Construction Services Association, 80 factors causing project delays were obtained based on previous studies. The distribution of questionnaires aimed to determine the level of influence of risk factors causing project delays; a closed questionnaire was used, where answer choices had been determined in advance, and respondents were given the opportunity to choose the most appropriate answer. ^63^ For data processing, we used a validity test, a reliability test, and descriptive analysis.

The questionnaire was composed of two parts: questionnaire A and questionnaire B. Questionnaire A concerned the characteristics of respondents, and questionnaire B concerned the level of influence of factors causing project delays. Assessment of the level of influence of 80 project delay risk factors was carried out using a Likert scale, which consists of five points as defined in previous studies ( *e.g.*, References 31, 50, 64). The Likert scale has previously been used to measure the perceptions of respondents about social events ^65^ ^–^ ^67^ and can be seen in Table 2.

**Table 2.** Likert scale.

No.	Category	Score
1	Very high influence	5
2	High influence	4
3	Medium influence	3
4	Low influence	2
5	Very low influence	1

### Data collection

The location of research was carried out in Central Aceh Regency, this was done because considering the very complex condition of Central Aceh Regency, the existence of socio-cultural diversity, high inflation rates, low public education, frequent disasters, community economic weakness, geographical location, socio-political conflicts, and the economic crisis. ^33^ Construction projects in Central Aceh Regency often experience delays, ^1^ and it is necessary to conduct research to identify and analyze the factors causing delays in construction projects in Central Aceh Regency. The data collected for this research were questionnaire data, from questionnaire tools distributed to respondents, namely contractor companies located in Central Aceh District. The collection of data was carried out over two months by the researchers. Primary data collection was done by distributing questionnaires, the questionnaire is a data collection technique that is done by giving a set of written questions to the respondents to be answered. The distribution of questionnaire was given directly to the respondents. The type of questionnaire used was a closed questionnaire, which is a questionnaire whose answers have been provided, so that respondents only need to choose the appropriate answer. ^65^ This questionnaire was distributed to 47 contractor companies. Secondary data was obtained from the office of the Indonesian National Construction Implementing Association, namely data on 53 contractor companies in Central Aceh Regency to be surveyed, related to the qualifications of each company, as well as company addresses. This study used probability sampling, namely simple random sampling in distributing questionnaires. Simple random sampling technique is a technique consisting in taking samples randomly from members of the population. ^68^ The targeted respondents were contractors from the Central Aceh Regency, which has a population of 53 contractor companies. The experimental procedures were approved by the Institutional Review Board at Syiah Kuala University (IRB protocol number 99). All of these experimental methods were carried out in accordance with the regulations of the Institutional Review Board of Syiah Kuala University in Indonesia, and all participants gave their informed consent. The total sample size was 47 companies, calculated from the total population with an inaccuracy allowance of 5%, then by using the Slovin formula. ^37^ The Slovin formula is used to determine the sampling size by calculation, ^68^ ^,^ ^69^ Data collection was performed by distributing questionnaires to respondents directly.


$$n=\frac{N}{1+\left(N\;x\;e^{2}\right)}$$ (1)

### Descriptive statistics

Descriptive statistics are used to collect, organize and process data to be presented and provide a clear picture, regarding a particular condition or event where the data is taken. Descriptive statistics are to present data clearly, in order to be taken or certain meanings. ^70^ Descriptive statistics provide an overview of the object under study through sample or population data without analyzing and making conclusions that apply to the public. ^71^ Quantitative descriptive research describes data in the form of numbers, and the size of the data includes the mean value, mode, and median. The size of the data deployment includes variance and standard deviation. ^72^ Descriptive statistical analysis determines the most influential factors on project delays, and uses mean and mode value, which is the data that appears most often.

## Results

### Validity test

The validity test is a tool to test whether each question item truly reveals the factors or indicators that need to be investigated. ^73^ Validity testing was performed by distributing the questionnaires to 20 respondents. A validity test was performed for each variable using Pearson's product moment analysis. The variable was considered valid if the rxy value was greater than the r-table value. The r-table value obtained was 0.288, with degrees of freedom (df) associated with or an error level of 0.05, in both directions. The question had a value greater than 0.288; therefore, the questionnaire was deemed feasible and valid.

### Reliability test

A reliability test was conducted to determine whether the questionnaire was reliable, with a coefficient of ≥ 0.6. If the value was above 0.60, the questionnaire was considered reliable and feasible to use. ^74^

As shown in Table 3, a reliability coefficient of 0.958 was obtained. This shows that the coefficient of Cronbach's alpha for the variable causing the delay was greater than 0.6. Therefore, the questionnaire was deemed to be reliable.

**Table 3.** Reliability test results.

No	Factors	Cronbach alpha	Conclusion
1	80	0.958	Reliable

### Respondents and company profiles

Questionnaires were distributed to 47 respondents; their characteristics are presented in Table 4.

**Table 4.** Characteristics of respondents.

No	Group	Frequency (N = 47)	Percentage (%)
1	Company period of activity		
	0–5 years	10	21.28%
	>6–10 years	11	23.40%
	>10–15 years	8	17.02%
	>15 years	18	38.30%
2	Number of projects handled		
	1–3	5	10.64%
	>3–6	4	8.51%
	>6–10	12	25.53%
	>10	26	55.32%
3	Estimated project duration each year		
	0–6 months	41	87.23%
	>6–12 months	6	12.77%
4	Graduates		
	High School/equivalen	0	0.00%
	Diploma	0	0.00%
	Bachelor	35	74.47%
	Master	12	25.53%
	Doctoral	0	0.00%
5	Long Experience in the construction field		
	0–2 years	0	0.00%
	3–5 years	17	36.17%
	6–8 years	20	42.55%
	>8 years	10	21.28%
6	Type of projects executed		
	New Projects	33	70.21%
	Renovation Projects	14	29.79%

Questionnaires were distributed to 47 respondents, and the results of distributing questionnaires on the characteristics of the respondents can be concluded based on the results of the research in Table 4. It was found that most companies, that is, 18 companies (38.30%) had over 15 years of experience in the construction sector, and the majority (26 companies, 55.32%) had handled several construction projects above 10. The majority *, i.e.* 41 companies (87.23%) had estimated project durations of 0–6 months per year. The majority of 35 respondents (74.47%) had a bachelor's level of education, the majority (20 respondents, 42.55%) had 6-8 years of experience in the construction industry, and most of the companies (33 companies, 70.21%) had implemented new projects.

### Level of influence of project delay factors

A descriptive analysis was used to determine the level of influence of the delay risk variable. The descriptive analysis uses the mode and mean values to determine the dominant delay factors. The results of the levels of the factors influencing project delays are shown in Table 5.

**Table 5.** Results of descriptive statistics on the level of influence of project delay factors.

No	Description of causes	n	Mode	Level of influence	Mean	Std. Deviation
1	Cost estimation inaccuracy	47	5	Very high	4.617	0.677
2	Implementation of new technologies	47	5	Very high	4.596	0.577
3	Tool malfunction	47	5	Very high	4.489	0.718
4	Workers quarrel	47	5	Very high	4.447	0.717
5	Poor project planning and controlling	47	5	Very high	4.426	0.683
6	Details, accuracy and conformity to specifications that are not appropriate	47	5	Very high	4.362	0.792
7	Increased work costs	47	5	Very high	4.340	0.915
8	Poor condition at locations and accessibility difficulty	47	5	Very high	4.340	0.867
9	The volume and type of material is not appropriate	47	4	High	4.319	0.556
10	Owner does not pay on time	47	4	High	4.319	0.862
11	Increase in material prices	47	4	High	4.298	0.805
12	Damage during shipping and storage material	47	4	High	4.298	0.623
13	Financial constraints on the contractor	47	4	High	4.298	0.657
14	Incompatibility of the use of costs with the progress of work	47	4	High	4.298	0.720
15	The lack of coordination/communication	47	4	High	4.298	0.749
16	Difficult access for heavy equipment to be used during the execution of the project site	47	4	High	4.277	0.971
17	Lack of worker availability	47	4	High	4.277	0.713
18	Decreased productivity	47	4	High	4.277	0.743
19	Profit target	47	4	High	4.255	0.988
20	Low worker productivity	47	4	High	4.255	0.642
21	Work permission overdue	47	4	High	4.255	0.736
22	Change in construction method	47	4	High	4.234	0.840
23	Lack of workers discipline	47	4	High	4.234	0.598
24	Material theft	47	4	High	4.213	0.623
25	Poor material planning & management	47	4	High	4.213	0.623
26	The amount of work that does not go according to plan	47	4	High	4.191	0.825
27	Substandard material quality	47	4	High	4.170	0.963
28	Unofficial charges	47	4	High	4.170	0.732
29	Limited material storage space	47	4	High	4.149	1.000
30	Incomplete equipment	47	4	High	4.149	0.955
31	Increased costs due to environmental safeguards	47	4	High	4.149	1.063
32	Damage of surrounding buildings due to project work	47	4	High	4.128	0.612
33	Not administrated in project documents	47	4	High	4.128	0.875
34	Power disturbances	47	4	High	4.128	0.769
35	Lack of availability of construction technology	47	4	High	4.106	0.699
36	Lack of project manager skill and experience	47	4	High	4.106	0.699
37	Lack of supervision of the schedule	47	4	High	4.106	0.729
38	Repairs due to repetitive work	47	4	High	4.106	0.866
39	Waste material handling	47	4	High	4.085	0.803
40	Did not predict unexpected costs	47	4	High	4.085	0.747
41	Delay Penalty	47	4	High	4.085	0.952
42	Availability of project financing sources (debtors) banks/third parties	47	4	High	4.085	0.905
43	Lack of cohesiveness of the work team	47	4	High	4.085	0.929
44	Supplier cannot fulfill material order	47	4	High	4.064	0.673
45	Delay in equipment mobilization	47	4	High	4.064	0.965
46	Incompetent and inexperienced engineer	47	4	High	4.064	0.734
47	Unaccurate construction method causes errors during project	47	4	High	4.043	0.908
48	Workers strike force	47	4	High	4.043	0.721
49	Difficulty to establish temporary facility	47	4	High	4.043	1.021
50	Lack of telecommunications network provision	47	4	High	4.043	0.751
51	Poor control and testing methods of quality	47	4	High	4.021	0.897
52	Lack of contractor skills and experience	47	4	High	4.021	0.737
53	Equipment misplacement	47	4	High	4.000	0.722
54	Negligence in checking the condition of the equipment	47	4	High	4.000	0.834
55	High equipment maintenance cost	47	4	High	4.000	0.808
56	The feasibility of the construction method	47	4	High	4.000	1.268
57	Lack of workers capability	47	4	High	4.000	0.834
58	Lack of supervision of subcontractors and suppliers	47	4	High	4.000	1.043
59	Changes in supplier/contractor performance	47	4	High	4.000	0.909
60	Small equipment capacity (small production)	47	4	High	3.979	1.113
61	Additional equipment rental costs	47	4	High	3.979	0.737
62	Planning changes that due to the results of site measurements and investigations	47	4	High	3.979	1.011
63	Wrong test method (lab error)	47	4	High	3.979	0.766
64	Changes to construction work due to implementation difficulty	47	4	High	3.979	0.821
65	Delay in material delivery	47	4	High	3.957	1.083
66	Equipment not in accordance with condition	47	4	High	3.936	1.223
67	Investors bankruptcy	47	4	High	3.915	1.195
68	Draft accuracy adjustment with the used construction methods	47	4	High	3.915	0.905
69	Loss of data and documents	47	4	High	3.915	0.974
70	No clear authority, duties, and responsibilities (unclear task delegation)	47	4	High	3.915	0.905
71	Productivity and efficiency decreased	47	4	High	3.894	0.890
72	Do not understand the procedure for using equipment	47	4	High	3.851	1.042
73	Ownership of the lease-purchase equipment	47	4	High	3.851	1.083
74	Lack of top management support	47	4	High	3.851	0.932
75	Poor equipment planning & management	47	4	High	3.830	0.842
76	Ownership of rental equipment	47	4	High	3.830	0.842
77	Availability of cash	47	4	High	3.787	1.250
78	Fuel scarcity	47	4	High	3.745	1.031
79	Ownership of proprietary equipment	47	4	High	3.723	0.877
80	Inefficient budgeting	47	3	Medium	3.681	1.024

Table 6 shows that of the 80 variables causing project delays, based on the respondents' opinions, there were eight risk factors in the very high influence category (Mode = 5), 71 factors of high influence category (Mode = 4), and one factor that belonged to the medium influence category (Mode = 3).

**Table 6.** Level influence based on number of items.

Level influence	Number of items
Very high influence	8
High influence	71
Medium influence	1
Low influence	0
Very low influence	0

**Figure 1.**
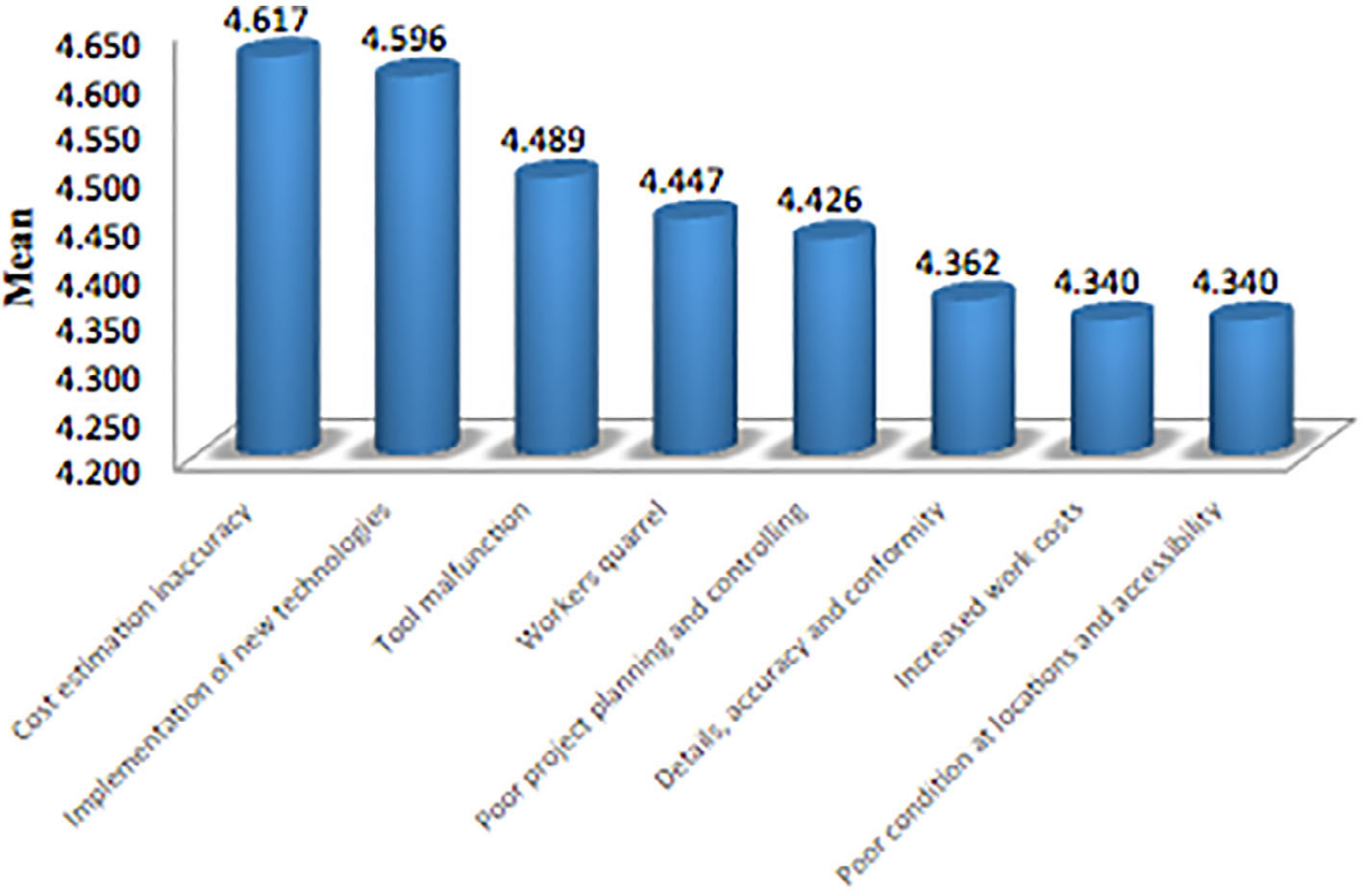
The mean values for the top eight factors are based on the results of the study.

Figure 1 Shows that the mean value for the top eight factors based on the results of the study was (1) cost estimation inaccuracy, (2) implementation of new technologies, (3) tool malfunction, (4) workers quarrel, (5) poor project planning and controlling, (6) details, accuracy and conformity to specifications that are not appropriate. (7) increased work costs, (8) poor condition at locations, and accessibility difficulty.

Figure 1 Shows that the mean value for the top eight factors based on the results of the study was cost estimation inaccuracy (4.617), implementation of new technologies (4.596), tool malfunction (4.489), workers quarrel (4.447), poor project planning and controlling (4.426), details, accuracy and conformity to specifications that are not appropriate (4.362), increased work costs (4.340), poor condition at locations, and accessibility difficulty (4.340).

**Figure 2.**
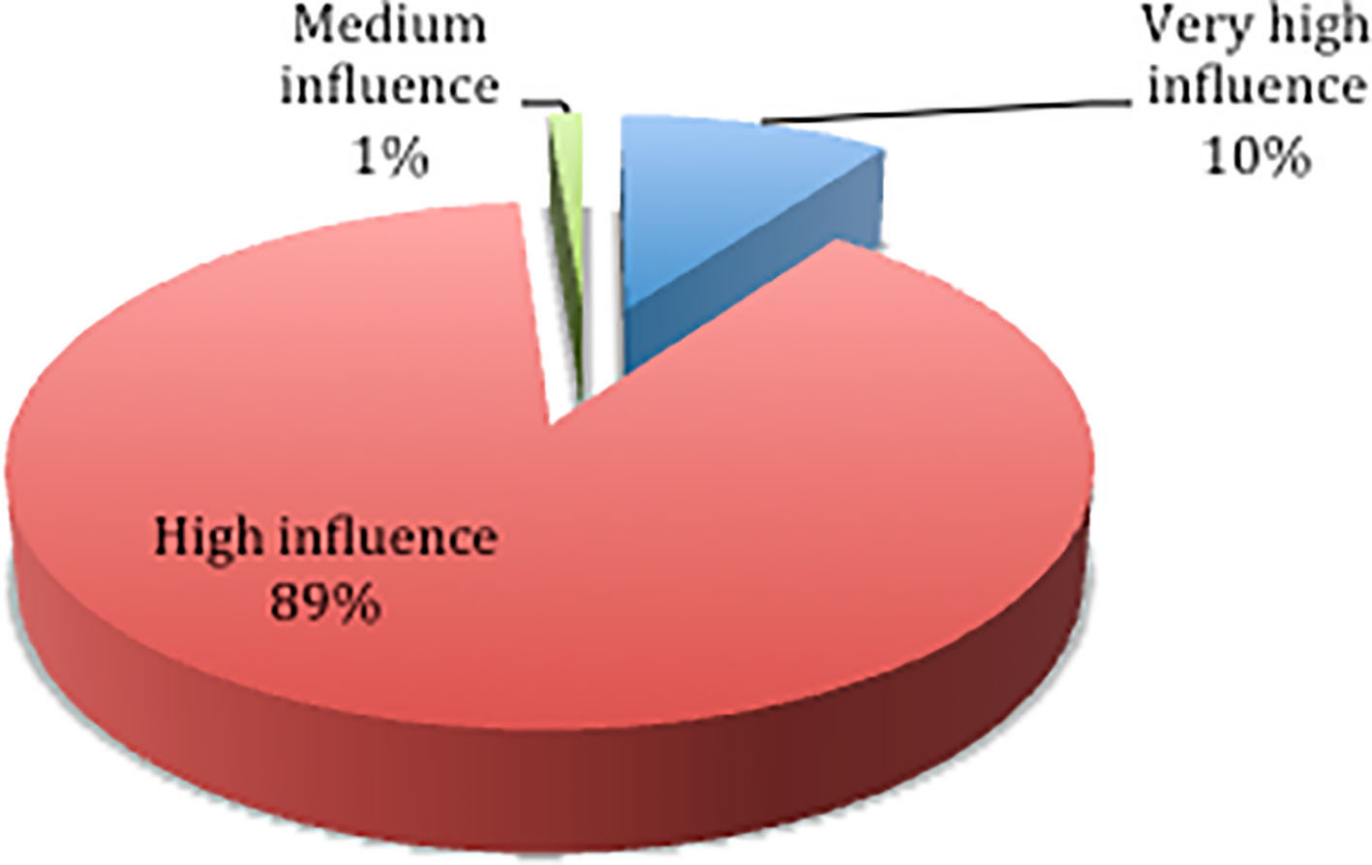
Percentage level of influence.

Based on the results of the questionnaire distribution, Figure 2 shows that 89% of respondents chose the high influence category, 10% chose the very high influence category, and 1% chose the medium influence category. The results of the descriptive statistics on the influence level of each delay factor are shown in Table 4.

## Discussion

The research results discussed in accordance with the research objectives are as follows;

1). Identify the risk factors that cause delays in construction projects.

The results showed that 80 factors of project delay were obtained based on previous studies, which were categorized into seven main factors are: (1) Material consists of 10 indicators; (2) Equipment consists of 17 indicators; (3) Financial consists of 14 indicators; (4) Construction Method consists of 11 indicators; (5) Workers consists of 8 indicators; (6) Contractor Management consists of 9 indicators; (7) Operational consists of 11 indicators.

2). Determine those particular risk factors that have a greater influence on construction projects.

The main factors causing project delays in Central Aceh District based on the mean values for the top eight factors are described as follows:

*Factor 1: Cost estimation inaccuracy*

Cost estimation inaccuracies can result in delays and losses. ^40^ Cost estimation is the calculation of the costs required to complete an activity or work by the requirements or contract, if the cost calculation is not correct, the risk increases and can cause losses to the project. Therefore, the contractor company is required to do an accurate cost estimate to avoid the risk. The main risk and uncertainty factor in a project is the estimated cost. ^38^ Errors in calculating cost estimates occur because the contractor it difficult to obtain adequate project document data, there are discrepancies and there is no clarity between the drawings and the work plan and requirements. The short time given can also result in contractors making mistakes in calculating project costs. The project cost estimator is expected to be able to predict what uncertainty factors may arise in the implementation of construction that can cause losses in the future. Cost estimation is very important for control, as a standard to compare reality and plans during the project. Project cost performance is influenced by several factors, and one of them is the accuracy factor in the cost estimation process. To achieve accuracy in cost estimation, contractor companies need to identify and anticipate risks that may occur in the project cost estimation procedure. With the identification and anticipation of risk in the cost estimation process, it is expected that project cost performance will be better, and there will be no cost overruns caused by inaccurate cost estimates. Contracting companies need to choose estimators who are experienced and can predict what risks will arise in project implementation to get an accurate cost estimate.

*Factor 2: Implementation of new technologies*

Contractor companies have difficulty in developing new technological innovations and creativity which are the key to winning the competition and building resilience in the construction industry. ^75^ Mastery and utilization of technology are needed by construction industry players to compete globally. ^75^ The application of new technology is still not widely known and understood by the contractor company so as to hamper the implementation of the project, cause the project to fail or cannot run according to plans and losses, and can cause project delays. ^6^ ^,^ ^40^ Respondents are small contractor companies that are still lacking in training and knowledge about the application of new technology. The use of innovation and technology is needed in infrastructure development so that development costs become cheaper, better, and faster. The use of appropriate, effective, inexpensive, and environmentally friendly technology is also encouraged to create added value and sustainable development so that the benefits of infrastructure can be felt by future generations.

*Factor 3: Tool malfunction*

Damage to equipment is often experienced by contractors that can cause losses, and endanger workers. ^6^ ^,^ ^40^ ^,^ ^41^ The problem that often occurs is the age of the equipment; The equipment is damaged because it is too old, and also the operator is not skilled in operating heavy equipment. To avoid equipment damage, contractors and project managers should carry out routine and periodic maintenance so that the tools are more durable in operation. Damage to equipment can cause losses and delays in the project, damage to heavy equipment can result in unfinished work, targets not being achieved, and cost overruns. To avoid losses due to equipment damage, contractors and managers need to know the mistakes that can cause equipment damage. Machine operator skills must be built through training, especially in using and operating machines. Without the right skills and knowledge about the heavy equipment to be operated, the operator may make mistakes that may be unintentional, but can be fatal to the machine's performance. In addition to training for operator skills, every machine must be equipped with a manual. Maintenance is something that must be done by the company in taking action to prevent damage. Regular maintenance can reduce the risk of damage. To avoid errors that can cause damage to heavy equipment, contractors and project managers need to carry out established procedures, as well as to check and maintain machines regularly.

*Factor 4: Workers quarrel*

Conflicts often occur between contractors, workers, and planner consultants. Conflicts that occur in project implementation are triggered by misuse of materials, equipment, design errors, and ineffective communication. Human resources are one of the most influential factors in construction projects. Workers come from various tribes who have different regional languages, this can also cause conflict because of non-smooth communication between them, project managers are expected to be able to manage human resources well to avoid delays. If a project is not supported by qualified human resources, it will not provide maximum and satisfactory results. Improper use and the occurrence of human resource conflicts result in huge losses for contractors. Workers’ quarrels are a risk factor that can disrupt the project because if there is a fight between workers, project implementation will automatically stop and cause delays. ^6^ ^,^ ^41^ The method used to resolve conflicts that occur between workers and human resources on projects is that companies and project managers need to take a problem-solving approach, namely, discussing openly and directly using dialogue between the parties involved, identifying problems that cause conflict, seeking and collecting information on the causes of conflict, and analyzing various alternatives that are considered to be the best solution. ^76^ Conflict management is needed within the company, so that there are no bigger problems due to the conflict. Conflict management can improve the creativity and performance of workers, develop employee skills, train conflict resolution skills, and increase mutual respect.

*Factor 5: Poor project planning and controlling*

Planning and control errors are often done by contractors, such as planning and controlling errors in materials, human resources, equipment, time, and costs so that the project implementation is not following the plan, and the project results also tend not to run as expected. Project managers and companies need to plan and control projects properly so that projects run smoothly and avoid losses. Poor project planning and control can result in delays and losses for the contractors. Contracting companies have a significant influence on project delays. ^77^ ^,^ ^78^ The views of clients and contractors on the causes of delays differ as they tend to blame each other for unfortunate incidents. ^14^ ^,^ ^41^ ^,^ ^42^ The first step in starting a project is that the contractor company needs to ensure that the project planning process which includes the project scope, project schedule, project resources, and project costs are running well. As an important element in project management, project planning involves developing actions and schedules that will keep the project moving consistently when executed according to its plan. An important step in project planning is that the contractor selects the resources needed to achieve the desired project objectives. Project control is very important for contractors, especially in anticipating problems that arise in the field, so that project implementation is not disrupted and goes according to plan. This can be achieved by efficient supervision, making activity reports, and holding coordination meetings to discuss problems that arise in the field and find ways to solve these problems.

*Factor 6: Details, accuracy and conformity to specifications that are inappropriate*

Inappropriate specifications can hinder the implementation of construction projects. ^40^ In the implementation of construction projects, deviations in the quality of work often occur, either as a result of intentional or unintentional acts by the contractor company, such as the contractor not understanding the design, specifications, and project documents. Errors in specifications and designs made by consultants can affect the quality of a building, and deviations from the agreement on the quality and time of completion of the work usually carry the risk of fines, which in the end have an impact on project losses and delays. Before carrying out the project, the contractor must understand the building drawings, details and specifications so that the resulting product has good quality. Quality is the level of good or bad a product produced, and by predetermined specifications or suitability to needs. For contractor companies, specifications are guidelines in meeting the expectations of service users through the process of implementing activities at the work site, which are based on plan drawings and specifications. Plan drawings serve as guidelines for realizing aspects of the shape and dimensions of the building, while specifications serve as guidelines for realizing aspects of building quality. For the estimator, the specification is very important because it states the quality of the material to be used. The notation for certain materials is written/drawn the same even though the quality aspect is different, this can be a source of conflict if no written explanation represents the quality of the material.

*Factor 7: Increased work costs*

Errors in cost estimation by contracting companies can cause an increase in project costs, this is because the estimator is less experienced in predicting the risks that will arise in project implementation. Cost overruns often occur in a project because the project implementation costs are greater than the project budget planning that has been set at the initial stage (estimated), which can cause significant losses for the project contractor. An increase in work costs is one of the causes of project delays. ^6^ ^,^ ^43^ The increase in the cost of work needs to be considered because it involves the amount of investment that must be made by the owner, where the cost overrun is vulnerable to the risk of failure. Contractor companies are expected to be able to manage project costs well, and can overcome the risk of failure to minimize the possibility of cost overruns. Contractor companies need to control the cost as a final step of the project cost management process, which ensures that the use and expenditure must be in accordance with predetermined planning so that there is no increase in work costs. Project risks often lead to mismatches between planning and implementation in the field, this can result in delays and cost overruns. A construction project is a planning or design process, and specifications are converted into physical structures and facilities. This process involves the organization and coordination of all project resources such as labor, construction equipment, materials, supplies and facilities, funds, technology. Project managers are expected to be able to manage project resources effectively and efficiently. Basically, in the implementation of construction projects, there are many projects that experience cost overruns and time delays. Expansion of costs at the project implementation stage is highly dependent on the planning, coordination, and control of the contractor.

*Factor 8: Poor conditions at locations and accessibility difficulty*

The contractor had difficulty accessing the project site in Central Aceh District which is hilly, surrounded by mountains and rivers, making access to the project site quite difficult. The condition of the project site is quite bad and difficult to reach can affect project delays and has the potential to cause project failure ^6^ ^,^ ^48^ because of (1) a lack of initial information on field conditions, (2) contractors not conducting initial surveys, and (3) the work environment not being prepared, such as land clearing and acquisition, fresh air supply, and adequate lighting. ^79^ To avoid project delays and failures, it is expected that the contractor can collect information and conduct an initial survey regarding the condition of the project site before implementing the project such that the contractor can plan strategies for the project to run smoothly. Difficulty in accessing the project site can cause resource mobilization to be slow. The mobilization referred to in this case is the movement of incoming materials and equipment to the project site. This is greatly influenced by the provision of project roads and the delivery time of tools or materials, difficulty in accessing the work site is one of the factors that cause project delays.

## Conclusions

Based on the results of the study, 80 factors that cause project delays have been obtained based on previous studies, and of the 80 variables causing project delays, based on the respondents' opinions, there were eight risk factors in the very high influence category (Mode = 5), 71 factors of high influence category (Mode = 4), and one factor that belonged to the medium influence category (Mode = 3), and the most dominant factor based on the mean value was the cost estimation inaccuracy (4.617).

The Indonesian government is actively engaged in construction in various sectors to create prosperity and welfare for its people. However, there are still many obstacles to working on construction projects that are not in accordance with the planned schedule. One of these obstacles is delays in construction projects. Obstacles and risks often occur during project implementation, resulting in project delays and losses. Delay in the implementation of construction projects is one of the risks that often occurs in the implementation of construction projects, especially in developing countries. Project delays for contractors can cause time and cost losses because the profits expected by the contractor are reduced, the contractor does not obtain the expected profits, or there may even be no profits at all. For project owners, delays in completing work can cause losses. Various methods have been implemented to avoid the problems that result in delays and losses. Identifying the root causes of delays is an important first step in mapping the problems that can cause project delays. The correct solution or strategy to overcome delays will be easier to obtain if the project has a map of the main factors that can cause the project to experience delays in the schedule. In this study, 80 factors causing project delays were identified, of which eight main factors were categorized as having a very high influence (= 5) in causing project delays.

The findings of this study are useful for academics and construction practitioners with potentially deeper insights into the root causes of project schedule delays. The continuous expansion of knowledge and understanding of the importance (criticality) of the causes of delays will assist stakeholders in reducing the incidence of delays, lead to appropriate strategies, and can be used as comparisons or benchmarks in development planning; thus, by knowing the causes of these delays, the contractor can properly calculate these risks to avoid losses impacting the project. However, further research should be conducted with a wider study area to increase the number of respondents.

The contractor is expected to pay attention to the dominant factors causing delays in order to reduce losses due to the risk of delays that occur in project implementation, and to formulate appropriate actions or responses for each risk that occurs in the project. Therefore, construction companies need to carry out risk management in accordance with applicable regulations.

This study has limitations, namely, sampling was only conducted in the Central Aceh District, and the scope of the study is not wide enough; therefore, the results of the study cannot be generalized to a wider population. The results of this study are specific to Central Aceh Regency, and are not expandable to other regions in Indonesia. Thus, similar studies can be conducted in other districts, provinces, and cities in Indonesia, and the results of the research can be generalized to other regions. Further research is needed to increase the number of respondents such that the results are more comprehensive.

### Suggestions for future studies

Our future research will aim to determine the effects of delay factors on construction project costs using the ordinal logistic regression method. Future research will be conducted to determine the delay factors that have a significant effect on construction project costs. These delay factors are expected to serve as a reference for contractor companies carrying out construction projects such that they can avoid construction project losses.

## Data availability

### Underlying data

Zenodo: Raw data for the study of the f1000 manuscript entitled: Critical Delay Factors for Construction Project in Central Aceh District, Indonesia

This project contains the following underlying data:

• Raw Data(1).xlsx

### Extended data

Zenodo: Raw data for the study of the f1000 manuscript entitled: Critical Delay Factors for Construction Project in Central Aceh District, Indonesia

This project contains the following extended data:

• Questionnaire1.xlsx

Data are available under the terms of the Creative Commons Attribution 4.0 International license (CC-BY 4.0).

## References

[1] Syam F : Community Leader of Central Aceh Regency: If the Multiyear Project Is Canceled, We Can Ask for Separation. *Aceh Journal National Network.* 2020.(accessed Feb. 07, 2021). Reference Source

[2] Qazi A Quigley J Dickson A : Project Complexity and Risk Management (ProCRiM): Towards modelling project complexity driven risk paths in construction projects. *Int. J. Proj. Manag.* 2016;34:1183–1198. 10.1016/J.IJPROMAN.2016.05.008

[3] Gray CF Larson EW : *Project Management: The Managerial Process.* Singapore: McGraw-Hill;2000.

[4] Rauzana A : The effect of the risk factors on the performance of contractors in Banda Aceh, indonesia. *ARPN J. Eng. Appl. Sci.* 2016;11(15):9496–9502.

[5] Alsuliman JA : Causes of delay in Saudi public construction projects. *Alex. Eng. J.* 2019;58(2):801–808. 10.1016/j.aej.2019.07.002

[6] Aziz RF Abdel-Hakam AA : Exploring delay causes of road construction projects in Egypt. *Alex. Eng. J.* 2016;55(2):1515–1539. 10.1016/j.aej.2016.03.006

[7] Mahdi I Soliman E : Significant and top ranked delay factors in Arabic Gulf countries. *Int. J. Constr. Manag.* 2019;21(14):167–180. 10.1080/15623599.2018.1512029

[8] Mpofu B Godfrey E Moobela OC : Profiling causative factors leading to construction project delays in the United Arab Emirates. *Eng. Constr. Archit. Manag.* 2017;24(2):346–376. 10.1108/ECAM-05-2015-0072

[9] Al Amri T Marey-Pérez M : Towards a sustainable construction industry: Delays and cost overrun causes in construction projects of Oman. *J. Proj. Manag.* 2020;5:87–102. 10.5267/j.jpm.2020.1.001

[10] Rumimper R : Risk Analysis on Housing Construction Projects in Minahasa-North Regency. *Sci. J. Media Eng.* 2015;5(2):381–389.

[11] Yap JB Goay PL Woon YB : Revisiting critical delay factors for construction: Analysing projects in Malaysia. *Alex. Eng. J.* 2021;60(1):1717–1729. 10.1016/j.aej.2020.11.021

[12] Alwi S Hampson K : Identifying the important causes of delays in building construction projects. *Proceedings Ninth EastAsia-Pacific Conference on Structural Engineering and Construction, Bali, Indonesia.* 2003; pp.1–6.

[13] Abu Salem ZT Suleiman A : Risk factors causing time delay in the Jordanian construction sector. *Int. J. Eng. Res. Technol.* 2020;13(2):307–315. 10.37624/ijert/13.2.2020.307-315

[14] Yap JBH Skitmore M : Investigating design changes in Malaysian building projects. *Archit. Eng. Des. Manag.* 2018;14(3):218–238. 10.1080/17452007.2017.1384714

[15] Zarei B Sharifi H Chaghouee Y : Delay causes analysis in complex construction projects: a Semantic Network Analysis approach. *Prod. Plan. Control.* 2018;29(1):29–40. 10.1080/09537287.2017.1376257

[16] Shahsavand P Marefat A Parchamijalal M : Causes of delays in construction industry and comparative delay analysis techniques with SCL protocol. *Eng. Constr. Archit. Manag.* 2018;25(4):497–533. 10.1108/ECAM-10-2016-0220

[17] Gbahabo PT Ajuwon OS : Effects of project cost overruns and schedule delays in Sub- Saharan Africa. *Eur. J. Interdiscip. Stud.* 2017;3(2):46–58. 10.26417/EJIS.V7I2.P46-59

[18] Sambasivan M Deepak TJ Salim AN : Analysis of delays in Tanzanian construction industry: Transaction cost economics (TCE) and structural equation modelling (SEM) approach. *Eng. Constr. Archit. Manag.* 2017;24(2):308–325. 10.1108/ECAM-09-2015-0145

[19] Aibinu AA Jagboro GO : The effects of construction delays on project delivery in Nigerian construction industry. *Int. J. Proj. Manag.* 2002;20(8):593–599. 10.1016/S0263-7863(02)00028-5

[20] Wu Y Zhou J : Risk assessment of urban rooftop distributed PV in energy performance contracting (EPC) projects: An extended HFLTS-DEMATEL fuzzy synthetic evaluation analysis. *Sustain. Cities Soc.* 2019;47(1):101524. 10.1016/j.scs.2019.101524

[21] PMI: *A guide to the project management body of knowledge (PMBOK guide).* 6th ed. USA: Newton Square, USA: Project Management Institute;2017.

[22] Qazi A Dikmen I : From risk matrices to risk networks in construction projects. *IEEE Trans. Eng. Manag.* 2019;68(12):1449–1460. 10.1109/TEM.2019.2907787

[23] Ansah RH Sorooshian S : Effect of lean tools to control external environment risks of construction projects. *Sustain. Cities Soc.* 2017;32:348–356. 10.1016/J.SCS.2017.03.027

[24] Hwang B Ngo J Her PWY : Integrated Digital Delivery: Implementation status and project performance in the Singapore construction industry. *J. Clean. Prod.* 2020;262:121396. 10.1016/j.jclepro.2020.121396

[25] Eskander RFA : Risk assessment influencing factors for Arabian construction projects using analytic hierarchy process. *Alex. Eng. J.* 2018;57(4):4207–4218. 10.1016/j.aej.2018.10.018

[26] Rauzana A : Identification and Assessment of Risk Factors Affecting Construction Projects in North Aceh, Indonesia. *IOSR J. Bus. Manag.* 2016;18(09):72–77. 10.9790/487x-1809047277

[27] Rauzana A : The Influence of Uncertainty Variables on Cost Estimation Lesson Learned From Construction Industry in Indonesia. *Aust. J. Basic Appl. Sci.* 2015;9(April):380–385.

[28] Leu SS Chen AT Yang CH : A GA-based optimal model for construction time-cost trade-off. *Int. J. Proj. Manag.* 2001;19(1):47–58. 10.1016/S0263-7863(99)00035-6

[29] PMI: *Construction Extension to the PMBOK ^®^ Guide.* Project Management Institute, Inc.;2016.

[30] Stumpf GR : Schedule delay analysis. *Cost Eng. Arbor Then Morgant.* 2000;42(7):32–32.

[31] Assaf SA Al-Hejji S : Causes of delay in large construction projects. *Int. J. Proj. Manag.* 2006;24(4):349–357. 10.1016/j.ijproman.2005.11.010

[32] Suryatmojo H : Residents agree on the price of land acquisition for the Central Aceh hydropower project, this is the price. *Antara Aceh.* 2020.

[33] Central Aceh District Government: *Integrated Plan And Medium-Term Infrastructure Investment Plan, Central Aceh District, 2016-2020.* Central Aceh District;2020.

[34] Kasidi: *Risk Management.* Bogor: Ghalia Indonesia;2010.

[35] Rauzana A Dharma W : The knowledge and awareness of occupational health and safety requirements among civil engineering students in an Indonesian university.vol. 23(no.3): pp.210–215.2021.

[36] Suárez M García-Romero E Baz A : Smectites: The key to the cost overruns in the construction of the third set of locks of the Panama Canal. *Eng. Geol.* 2021;284:106036. 10.1016/j.enggeo.2021.106036

[37] Rauzana A Dharma W : Causes of delays in construction projects in the Province of Aceh, Indonesia. *PLoS One.* 2022;17(1):e0263337. 10.1371/journal.pone.0263337 35089971 PMC8797244

[38] Aidil M Rauzana A Muhammad N : A Study on Drinking Water Distribution Project in Banda Aceh. *J. Phys. Conf. Ser.* 2021;1933(1):012091. 10.1088/1742-6596/1933/1/012091

[39] Rauzana A Dharma W : The effectiveness of on-line learning at an Indonesian university during the Covid-19 pandemic: students’ perspectives. *World Trans. Eng. Technol. Educ.* 2022;20(1):71–75.

[40] Sangari F Tjakra J : Risk Analysis of Housing Construction Projects in Manado City. *Sci. J. Media Eng.* 2011;1(1):29–37.

[41] Aziz RF : Ranking of delay factors in construction projects after Egyptian revolution. *Alex. Eng. J.* 2013;52(3):387–406. 10.1016/j.aej.2013.03.002

[42] Al-Kharashi A Skitmore M : Causes of delays in Saudi Arabian public sector construction projects. *Constr. Manag. Econ.* 2009;27(1):3–23. 10.1080/01446190802541457

[43] Rodrigues-da-Silva LH Crispim JA : The project risk management process, a preliminary study. *HCIST J.* 2014;16:943–949. 10.1016/j.protcy.2014.10.047

[44] Azhari T . . Aulia, and Majid I : Risk Factors Affecting Contractor Performance in Implementation of Infrastructure Projects in Aceh Jaya District. *Spektran J.* 2014;3(1):1–14.

[45] Khodeir LM El Ghandour A : Examining the role of value management incontrolling cost overrun [application on residential construction projects in Egypt]. *Ain. Shams. Eng. J.* 2019;10(3):471–479. 10.1016/j.asej.2018.11.008

[46] Khodeir L Mohamed AH : Identifying the latest risk probabilities affecting construction projects in Egypt according to political and economic variables. From January 2011 to January 2013. *HBRC J.* 2015;11:129–135. 10.1016/j.hbrcj.2014.03.007

[47] Nepal MP Park M Son B : Effects of schedule pressure on construction performance. *J. Constr. Eng. Manag.* 2006;132(2):182–188. 10.1061/(ASCE)0733-9364(2006)132:2(182)

[48] Muka I : Risk Analysis in the Sulawesi Denpasar Road Basement Parking Development Project. *J. Civ. Eng. Commun. Media.* 2012;19(2):155–165. 10.14710/mkts.v19i2.8425

[49] Toor S-U-R Ogunlana S : Problems causing delays in major construction projects in Thailand. *Constr. Manag. Econ.* 2008;26(4):395–408. 10.1080/01446190801905406

[50] Le-Hoai L Lee YD Lee JY : Delay and cost overruns in Vietnam large construction projects: A comparison with other selected countries. *KSCE J. Civ. Eng.* 2008;12(6):367–377. 10.1007/s12205-008-0367-7

[51] Amoah C Pretorius L : Evaluation of the impact of risk management on project performance in small construction firms in South Africa: The case study of construction systems. *J. Eng. Des. Technol.* 2020;18(3):611–634. 10.1108/JEDT-06-2018-0098

[52] Ramli S : *Risk Management in the Perspective of K3 OHS Risk Management.* Jakarta: Dian Rakyat;2010.

[53] Dikmen I Birgonul MT : A review of international construction research: Ranko Bon’s contribution. *J. Constr. Manag. Econ.* 2006;24(7):725–733. 10.1080/01446190600601909

[54] Molenaar KR : Programmatic cost risk analysis for highway megaprojects. *J. Constr. Eng. Manag.* 2005;131(3):343–353. 10.1061/(ASCE)0733-9364(2005)131:3(343)

[55] Larsen J Shen G Lindhard S : Factors affecting schedule delay, cost overrun, and quality level in public construction projects. *J. Manage. Eng.* 2016;32(1). 10.1061/(ASCE)ME.1943-5479.0000391

[56] Almamlook R Bzizi M Al-Kbisbeh M : Factors Affecting LaborProductivity in the Construction Industry. *Am. J. Env. Sci Eng.* 2020;4(2):24–30. 10.11648/j.ajese.20200402.13

[57] Abusafiya H Suliman S : Causes and effects of cost overrun on constructionproject in Bahrain: Part I (ranking of cost overrun factors and risk mapping). *Mod. Appl. Sci.* 2017;11(7):20. 10.5539/mas.v11n7p20

[58] Memon A Akhund M Laghari A : Adoptability ofLean Construction Techniques in Pakistan’s Construction Industry. *Civ. Eng. J.* 2018;4(10):28–37. 10.28991/cej-03091162

[59] Mahamid I : Risk matrix for factors affecting time delay in road construction projects: owners’ perspective. *Eng. Constr. Archit. Manag.* 2011;18(6):609–617. 10.1108/09699981111180917

[60] Mezher TM Tawil W : Causes of delays in the construction industry in Lebanon. *Eng. Constr. Archit. Manag.* 1998;5(3):252–260. 10.1108/eb021079

[61] Senouci A Ismail A Eldin N : Time Delay and Cost Overrun in Qatari Public Construction Projects. *Procedia Eng.* 2016;164(June):368–375. 10.1016/j.proeng.2016.11.632

[62] Koushki PA Al-Rashid K Kartam N : Delays and cost increases in the construction of private residential projects in Kuwait. *Constr. Manag. Econ.* 2005;23(3):285–294. 10.1080/0144619042000326710

[63] Nasution S : *Research Methods (Scientific Research).* Jakarta: Bumi Aksara; 6th ed. 2003.

[64] Bagaya O Song J : Empirical study of factors influencing schedule delays of public construction projects in Burkina Faso. *J. Manag. Eng.* 2016;32(5):05016014. 10.1061/(ASCE)ME.1943-5479.0000443

[65] Sugiyono: *Combined Research Methods (Mixed Methods).* Bandung: Alfabeta;2013.

[66] Riduwan and Sunarto: *Introduction to Statistics for Research: Education, Social, Communication, Economics, and Business.* Bandung: Alfabeta;2014.

[67] Vagias WM : *Likert-type scale response anchors. Clemson International Institute for Tourism & Research Development.* Department of Parks, Recreation and Tourism Management, Clemson University;2006.

[68] Sugiyono: *Combination Research Methods.* Bandung: Alfabeta;2015.

[69] Noor J :2012; *Research Methodology.* Jakarta: Kencana Prenada Media Group.

[70] Hasan I : *Research Data Analysis with Statistics.* Jakarta: PT Bumi Aksara;2004.

[71] Jaya I : *Application of Statistics for Educational Research.* jakarta, Indonesia: Prenadamedia Group;2019.

[72] Kaur P Stoltzfus J Yellapu V : Descriptive statistics. *Int. J. Acad. Med.* 2018;4(1):60–63. 10.4103/IJAM.IJAM_7_18

[73] Arikunto S : *Research Management.* Jakarta: Rineka Cipta;2013.

[74] Sekaran U : *Research methods for business: A skill building approach.* 4th ed. John Wiley & Sons;2006.

[75] Kusumawanti R : *Construction Industry Needs New Technology.* Portonews;2019.(accessed Jun. 07, 2021). Reference Source

[76] Susila H : Conflict Handling Methods In The Implementation Of Building Construction Projects In Surakarta. *J. Civ. Archit. Eng.* 2012;12(16):6–10.

[77] Mydin MAO Sani NMM Taib NM : Imperative causes of delays in construction projects from developers’ outlook. 2014. 10.1051/matecconf/20141006005

[78] Rao PB Camron CJ : Causes of delays in construction projects: A case study. *Int. J. Curr. Res.* 2014;6(6):7219–7222.

[79] Price D Andy K : Causes Leading to Poor site Coordination in Building Projects. *Organ. Technol. Manag. Constr. - An Int. J.* 2010;2(2):167–172.

