# Supplementary material for: Critical delay factors for construction projects in Central Aceh District, Indonesia
Source: F1000Res. 2022 Sep 29;11:474. Originally published 2022 Apr 29. [Version 3] doi: 10.12688/f1000research.110024.3 (PMC9860196; doi:10.12688/f1000research.110024.3)
Supplement: Peer review history

# Peer review: Reviewer response for version 3

**DOI:** 10.5256/f1000research.138988.r151912

**Published:** 2023-01-20

**Authors:** Atasya Osmadi

**Affiliations:** 1 School of Housing Building and Planning, Universiti Sains Malaysia, George Town, Malaysia

Approved for indexing as authors have improved the article as recommended

Is the work clearly and accurately presented and does it cite the current literature?

Partly

If applicable, is the statistical analysis and its interpretation appropriate?

Partly

Are all the source data underlying the results available to ensure full reproducibility?

Partly

Is the study design appropriate and is the work technically sound?

Partly

Are the conclusions drawn adequately supported by the results?

Partly

Are sufficient details of methods and analysis provided to allow replication by others?

Partly

Reviewer Expertise:

Construction Project Management

I confirm that I have read this submission and believe that I have an appropriate level of expertise to confirm that it is of an acceptable scientific standard.

# Peer review: Reviewer response for version 3

**DOI:** 10.5256/f1000research.138988.r151911

**Published:** 2022-10-13

**Authors:** Christopher Amoah

**Affiliations:** 1 Department of Quantity Surveying and Construction Management, University of the Free State, Bloemfontein, South Africa

The authors have significantly made changes to the paper as suggested.

Is the work clearly and accurately presented and does it cite the current literature?

Partly

If applicable, is the statistical analysis and its interpretation appropriate?

Yes

Are all the source data underlying the results available to ensure full reproducibility?

Yes

Is the study design appropriate and is the work technically sound?

No

Are the conclusions drawn adequately supported by the results?

Partly

Are sufficient details of methods and analysis provided to allow replication by others?

Partly

Reviewer Expertise:

Construction project management

I confirm that I have read this submission and believe that I have an appropriate level of expertise to confirm that it is of an acceptable scientific standard.

# Peer review: Reviewer response for version 2

**DOI:** 10.5256/f1000research.137176.r147653

**Published:** 2022-09-15

**Authors:** Atasya Osmadi

**Affiliations:** 1 School of Housing Building and Planning, Universiti Sains Malaysia, George Town, Malaysia

The authors have addressed some of the issues raised in the first review. Thank you for your effort to improve this article.

 There are still lack of explanation in some parts of the article. Most are written with broad statements. For example in **Abstract, **what does it mean by distinctive characteristics and are very complex? Please explain further. Also in **Introduction, **what does it mean by construction projects are dynamic and consist of limited resources. Do explain further and give examples. Check throughout the article and improve your explanation. Avoid giving broad statements.

 There is also lack of explanation or linking sentences to why research from Jordan, Nigeria, Saudi Arabia & Palestine is being included here. Is there lack of research on this area from other countries or do they share certain similarities with Central Aceh? Do improve your discussion.

 Clearly explain the significant of mode value and why it is sufficient enough for this research article.

 Do explain your findings for the two objectives with clear headings in the discussion. 

 The sentence 'The results showed that 80 factors of project delay were obtained based on previous studies, and of the 80 variables causing project delays, based on the respondents' opinions, there were eight risk factors in the very high influence category (Mode = 5), 71 factors of high influence category (Mode = 4), and one factor that belonged to the medium influence category (Mode = 3)' is written twice in discussion and conclusion. Consider to delete one sentence. Please check throughout article and avoid writing the same sentence twice.

Is the work clearly and accurately presented and does it cite the current literature?

Partly

If applicable, is the statistical analysis and its interpretation appropriate?

Partly

Are all the source data underlying the results available to ensure full reproducibility?

Partly

Is the study design appropriate and is the work technically sound?

Partly

Are the conclusions drawn adequately supported by the results?

Partly

Are sufficient details of methods and analysis provided to allow replication by others?

Partly

Reviewer Expertise:

Construction Project Management

I confirm that I have read this submission and believe that I have an appropriate level of expertise to confirm that it is of an acceptable scientific standard, however I have significant reservations, as outlined above.

# Response

**Published:** 2022-09-22

**Authors:** Anita Rauzana

**Affiliations:** Universitas Syiah Kuala, Indonesia

**REVIEWER COMMENT** **:**

 The authors have addressed some of the issues raised in the first review. Thank you for your effort to improve this article.

 There are still lack of explanation in some parts of the article. Most are written with broad statements. For example in **Abstract, **what does it mean by distinctive characteristics and are very complex? Please explain further.

**RESPONSE :** **Thank you very much. We agree with the reviewer, and we have fixed it by using a more specific statement.**

**REVIEWER COMMENT** **:**

 Also in **Introduction, **what does it mean by construction projects are dynamic and consist of limited resources. Do explain further and give examples. Check throughout the article and improve your explanation. Avoid giving broad statements.

**RESPONSE :** **Thank you very much. We agree with the reviewer, and we have fixed it by using a more specific statement.**

**REVIEWER COMMENT** **:**

 There is also lack of explanation or linking sentences to why research from Jordan, Nigeria, Saudi Arabia & Palestine is being included here. Is there lack of research on this area from other countries or do they share certain similarities with Central Aceh? Do improve your discussion.

**RESPONSE :** **Thank you very much, it has been fixed and has added discussion in the introduction at paragraph 8 line 1.**

**REVIEWER COMMENT** **:**

 Clearly explain the significant of mode value and why it is sufficient enough for this research article.

**RESPONSE :** **Thank you very much,** **Mode value is the value that appears most often in statistical data. The mode is also the majority value or the value with the highest frequency, to strengthen the results of the study, we have added the mean and standard deviation values for each factor and have obtained the highest mean value for the eight largest rankings. This explanation has been added in Methods and Results.**

**REVIEWER COMMENT** **:**

 Do explain your findings for the two objectives with clear headings in the discussion. 

**RESPONSE :** **Thank you very much. we agree with the reviewer, and we have repair the discussion based on 2 main** **research objectives** **with clear headings** **.**

**REVIEWER COMMENT** **:**

 The sentence 'The results showed that 80 factors of project delay were obtained based on previous studies, and of the 80 variables causing project delays, based on the respondents' opinions, there were eight risk factors in the very high influence category (Mode = 5), 71 factors of high influence category (Mode = 4), and one factor that belonged to the medium influence category (Mode = 3)' is written twice in discussion and conclusion. Consider to delete one sentence. Please check throughout article and avoid writing the same sentence twice.

**RESPONSE :** **Thank you very much. We agree with the reviewer and we have fixed it**

# Peer review: Reviewer response for version 2

**DOI:** 10.5256/f1000research.137176.r147652

**Published:** 2022-09-06

**Authors:** Christopher Amoah

**Affiliations:** 1 Department of Quantity Surveying and Construction Management, University of the Free State, Bloemfontein, South Africa

The author failed to address all the issues raised in the first review, which is not acceptable. The author should discuss the findings based on the two main project objectives with clear headings as suggested. Currently, the influence levels of the factors are passively explained without talking about their implications for project implementers and managers.

 The author should redo the following:

**Result and discussion **

- The author should calculate the mean and standard deviation values for all the factors to see if the top 8 factors mentioned will suffice. 
- The factors must be arranged in order of importance based on the mean values. I even suggest P-Values or factors analysis is done for the 80 factors.

-  The discussion should be based on the 2 main research objectives, namely
  - (1) identify the risk factors that cause delays in construction projects
  - (2) determine those particular risk factors that have a greater influence on construction projects.

Is the work clearly and accurately presented and does it cite the current literature?

Partly

If applicable, is the statistical analysis and its interpretation appropriate?

Yes

Are all the source data underlying the results available to ensure full reproducibility?

Yes

Is the study design appropriate and is the work technically sound?

No

Are the conclusions drawn adequately supported by the results?

Partly

Are sufficient details of methods and analysis provided to allow replication by others?

Partly

Reviewer Expertise:

Construction project management

I confirm that I have read this submission and believe that I have an appropriate level of expertise to state that I do not consider it to be of an acceptable scientific standard, for reasons outlined above.

# Response

**Published:** 2022-09-22

**Authors:** Anita Rauzana

**Affiliations:** Universitas Syiah Kuala, Indonesia

**REVIEWER COMMENT** **:**

 The author failed to address all the issues raised in the first review, which is not acceptable. The author should discuss the findings based on the two main project objectives with clear headings as suggested. Currently, the influence levels of the factors are passively explained without talking about their implications for project implementers and managers.

**RESPONSE :** **Thank you very much. We agree with the reviewer, and we have added an explanation by discussing the implications for project implementers and managers, and discussion based on 2 main research objectives** **with clear headings** **.**

**REVIEWER COMMENT** **:**

**Result and discussion**

 The author should calculate the mean and standard deviation values for all the factors to see if the top 8 factors mentioned will suffice. 

**RESPONSE :** **Thank you very much. We agree with the reviewer, and we have calculated the mean and** **standard deviation** **values for all factors in Table 5.**

**REVIEWER COMMENT** **:**

 The factors must be arranged in order of importance based on the mean values. I even suggest P-Values or factors analysis is done for the 80 factors.

**RESPONSE :** **Thank you very much. We agree with the reviewer, and we have** **arranged** **the factors** **based on the** **mean values.**

**REVIEWER COMMENT** **:**

  The discussion should be based on the 2 main research objectives, namely

 (1) identify the risk factors that cause delays in construction projects

 (2) determine those particular risk factors that have a greater influence on construction projects.

**RESPONSE : Thank you very much. We agree with the reviewer, and we have repair the discussion based on 2 main research objectives.**

# Peer review: Reviewer response for version 1

**DOI:** 10.5256/f1000research.121592.r143751

**Published:** 2022-08-01

**Authors:** Atasya Osmadi

**Affiliations:** 1 School of Housing Building and Planning, Universiti Sains Malaysia, George Town, Malaysia

**Abstract**

- There is a lack in explaining why this article is interesting.

**Introduction**

- The problem or issues to why this research is conducted in Central Aceh District is not properly discussed. The authors mainly discussed the  project delay factors in other countries and not discussing on the case study area/country. 

**Methodology**

- The author claimed that secondary data obtained from the literature related to this particular type of research and yet there was no section on literature review and how the 80 factors were determined. The reviewer could not tell which articles is being referred by the authors to determine this 80 factors.
- The author should explain why the Central Aceh district was chosen for this research and where do they get the list or if there is any database for the 53 contractor companies. It is hard to tell if this research is significant without proper explanation

**Results**

- A Research Article should be no more than 20,000 words but this research article has only around 5000 word. Results should be provided as required from the journal's format which includes: Preliminary pilot testing and any changes implemented resulting from preliminary testing

**Conclusion/Discussion**

- There is lack of discussion in this section. Discussion based on the results should be here

Is the work clearly and accurately presented and does it cite the current literature?

Partly

If applicable, is the statistical analysis and its interpretation appropriate?

Partly

Are all the source data underlying the results available to ensure full reproducibility?

Partly

Is the study design appropriate and is the work technically sound?

Partly

Are the conclusions drawn adequately supported by the results?

Partly

Are sufficient details of methods and analysis provided to allow replication by others?

Partly

Reviewer Expertise:

Construction Project Management

I confirm that I have read this submission and believe that I have an appropriate level of expertise to confirm that it is of an acceptable scientific standard, however I have significant reservations, as outlined above.

# Response

**Published:** 2022-08-06

**Authors:** Anita Rauzana

**Affiliations:** Universitas Syiah Kuala, Indonesia

**ABSTRACT**

**REVIEWER COMMENT** **:** There is a lack in explaining why this article is interesting.

**RESPONSE: Thank you very much. We agree with the reviewer and we've added an explanation in the abstract of why this article is interesting.**

**INTRODUCTION**

**REVIEWER COMMENT** **:**

 The problem or issues to why this research is conducted in Central Aceh District is not properly discussed. The authors mainly discussed the project delay factors in other countries and not discussing on the case study area/country. 

**RESPONSE: Thank you very much. We agree with the reviewer and we have added an explanation of why this research was conducted Central Aceh District**

**METHODOLOGY**

**REVIEWER COMMENT**: The author claimed that secondary data obtained from the literature related to this particular type of research and yet there was no section on literature review and how the 80 factors were determined. The reviewer could not tell which articles is being referred by the authors to determine this 80 factors.

**RESPONSE: Thank you very much. We agree with the reviewer and we have added a literature review section and have defined the literature referring to the 80 factors of delay in Table 1 in the Literature Review section.**

**REVIEWER COMMENT**: The author should explain why the Central Aceh district was chosen for this research and where do they get the list or if there is any database for the 53 contractor companies. It is hard to tell if this research is significant without proper explanation

**RESPONSE: Thank you very much. We agree with the reviewer and we have explained why the Central Aceh district was chosen for this research, and also explained where to get the list of 53 contractor companies.**

**RESULTS**

**REVIEWER COMMENT**: A Research Article should be no more than 20,000 words but this research article has only around 5000 word. Results should be provided as required from the journal's format which includes: Preliminary pilot testing and any changes implemented resulting from preliminary testing

**RESPONSE: Thank you very much. We agree with the reviewer and we have added words to this research article and it has reached 11068 words, and we have separated the results section from the discussion section.**

**CONCLUSION/DISCUSSION**

**REVIEWER COMMENT**: There is lack of discussion in this section. Discussion based on the results should be here

**RESPONSE: Thank you very much. We agree with the reviewer and we have added a discussion section based** **on research results**

# Peer review: Reviewer response for version 1

**DOI:** 10.5256/f1000research.121592.r142319

**Published:** 2022-07-20

**Authors:** Christopher Amoah

**Affiliations:** 1 Department of Quantity Surveying and Construction Management, University of the Free State, Bloemfontein, South Africa

**Abstract**

- The abstract omitted the implication of the study and the value of the paper
- The author can state the factors without stating the number of participants and percentages in the result section.

**Keywords** should be arranged in an alphabetical order

**Introduction**

 The introduction is not well-written, and the research objective is not clearly stated. The authors did not discuss problem implementation challenges in the case study district that are needed to be addressed by this study. The authors discussed project delay factors in other countries without focusing on the case study area/country. The author must address these deficiencies.

**Literature**

 This section was omitted. The author should discuss the following topics

1. Causes of project delays
2. Effects of project delays on stakeholders
3. Project risk factors
4. The description of the case study district

**Methodology **

- The author claimed to have studied literature, yet there was no literature review section
- The author should explain how the 80 factors were arrived at.
- The author should back the efficacy of the sample size used for this study with some authorities in the literature.
- The author should explain the procedures used in collecting the data
- The author should explain why the Central Aceh district was chosen for the study
- Table 2 should be removed from this section.

**Result and discussion **

- The demographic data should include experience in the construction industry, educational level and nature of projects executed
- I suggest the author calculate the mean and standard deviation values for all the factors to see if the top 8 factors mentioned will suffice.
- Create another column in table 5 and indicate the influence levels
- The discussions of the top factors are shallow. The authors should also discuss the implications of the findings
- The author should separate results from discussions
- The discussion should be based on the 2 main research objectives, namely
  1. identify the risk factors that cause delays in construction projects
  2. determine those particular risk factors that have a greater influence on construction projects.

**Conclusion**

 Recommendations should be given to the concerned stakeholders

**Communication**

 The quality of communication is good, but proofreading is required

Is the work clearly and accurately presented and does it cite the current literature?

Partly

If applicable, is the statistical analysis and its interpretation appropriate?

Yes

Are all the source data underlying the results available to ensure full reproducibility?

Yes

Is the study design appropriate and is the work technically sound?

No

Are the conclusions drawn adequately supported by the results?

Partly

Are sufficient details of methods and analysis provided to allow replication by others?

Partly

Reviewer Expertise:

Construction project management

I confirm that I have read this submission and believe that I have an appropriate level of expertise to state that I do not consider it to be of an acceptable scientific standard, for reasons outlined above.

# Response

**Published:** 2022-08-06

**Authors:** Anita Rauzana

**Affiliations:** Universitas Syiah Kuala, Indonesia

**ABSTRACT**

**REVIEWER COMMENT** **:**

 The abstract omitted the implication of the study and the value of the paper

**RESPONSE: Thank you very much. We agree with the reviewer and we have added study implication in the abstract**

**REVIEWER COMMENT** **:**

 The author can state the factors without stating the number of participants and percentages in the result section.

**RESPONSE: Thank you very much. We have stated the factors without stating the number of participants and percentages in the result section.**

**REVIEWER COMMENT**:

 Keywords should be arranged in an alphabetical order

**RESPONSE: Thank you very much. We agree with the reviewer and we've fixed the writing of keywords in alphabetical order**

**INTRODUCTION**

**REVIEWER COMMENT**: The introduction is not well-written, and the research objective is not clearly stated.

**RESPONSE**: **Thank you very much.** **We have corrected the writing of the Introduction and the research objectives have been clearly stated.**

**REVIEWER COMMENT**: The authors did not discuss problem implementation challenges in the case study district that are needed to be addressed by this study. The authors discussed project delay factors in other countries without focusing on the case study area/country. The author must address these deficiencies.

**RESPONSE: Thank you very much. We agree with the reviewer and we have written a discussion of the challenges of implementing the problems in the case study districts that need to be addressed by this research.**

**LITERATURE**

**REVIEWER COMMENT**:

 This section was omitted. The author should discuss the following topics

 1. Causes of project delays

 2. Effects of project delays on stakeholders

 3. Project risk factors

 4. The description of the case study district

**RESPONSE: Thank you very much. We agree with the reviewer, we have added Literature Review to discuss the following topics:**

**1. Causes of project delays**

**2. Effects of project delays on stakeholders**

**3. Project risk factors**

**4. The description of the case study district**

**METHODOLOGY**

**REVIEWER COMMENT** **: **The author claimed to have studied literature, yet there was no literature review section

**RESPONSE:** **Thank you very much. We agree with the reviewer, we have added Literature Review**

**REVIEWER COMMENT** **: **The author should explain how the 80 factors were arrived at

**RESPONSE:** **Thank you very much, we have** **explained how the 80 factors were arrived at**

**REVIEWER COMMENT**: The author should back the efficacy of the sample size used for this study with some authorities in the literature.

**RESPONSE:** **Thank you very much, we have added literature to support the efficacy of the sample size used for this study by several authorities in the literature.**

**REVIEWER COMMENT**:

 The author should explain the procedures used in collecting the data

**RESPONSE:** **Thank you very much, w** **e have added an explanation of the procedure** **used in collecting the data**

**REVIEWER COMMENT**:

 The author should explain why the Central Aceh district was chosen for the study

**RESPONSE:** **Thank you very much** **, we have added an explanation of why the Central Aceh district was chosen for the study**

**REVIEWER COMMENT**:

 Table 2 should be removed from this section.

**RESPONSE:** **Thank you very much. we agree with the reviewer, we already deleted Table 2 in the methodology section.**

**RESULT AND DISCUSSION**

**REVIEWER COMMENT**: The demographic data should include experience in the construction industry, educational level, and nature of projects executed

**RESPONSE:** **Thank you very much** **, we've added experience in the construction industry, level of education, and nature of projects executed to the demographic data.**

**REVIEWER COMMENT**: I suggest the author calculate the mean and standard deviation values for all the factors to see if the top 8 factors mentioned will suffice.

**RESPONSE: Thank you very much. We appreciate the suggestion given by the reviewer to calculate the mean and standard deviation values for all the factors to see if the top 8 factors mentioned will suffice. However, in this study, we use the mode value to obtain the most influential delay factors (Mode=5) on a construction project, where the results show that of the 80 variables causing project delays, based on the respondents' opinions, there were eight risk factors in the very high influence category (Mode = 5), 71 factors of high influence category (Mode = 4), and one factor that belonged to the medium influence category (Mode = 3).**

**REVIEWER COMMENT**:

 Create another column in table 5 and indicate the influence levels

**RESPONSE: Thank you very much. We have created a column in table 4 which shows the level of influence of the factors that have been made in Table 4, namely in the fifth column**

**REVIEWER COMMENT**: The discussions of the top factors are shallow. The authors should also discuss the implications of the findings.

**RESPONSE: Thank you very much. We have added a discussion of the implications of the research findings**

**REVIEWER COMMENT**:

 The author should separate results from discussions

**RESPONSE: Thank you very much. We have separated the results from the discussion**

**REVIEWER COMMENT**:

 The discussion should be based on the 2 main research objectives, namely:

1. identify the risk factors that cause delays in construction projects
2. determine those particular risk factors that have a greater influence on construction projects.

**RESPONSE: Thank you very much. We have added a discussion based on 2 main research objectives, namely:**

1. **Identify the risk factors that cause delays in construction projects**
2. **Determine those particular risk factors that have a greater influence on construction projects.**

**CONCLUSION**

**REVIEWER COMMENT**:

 Recommendations should be given to the concerned stakeholders

**RESPONSE: Thank you very much. We agree with the reviewer and we have added recommendations to** **concerned** **stakeholders in the conclusion section**

**COMMUNICATION**

**REVIEWER COMMENT**:

 The quality of communication is good, but proofreading is required

**RESPONSE: Thank you very much. We have proofread this article**
